# Engineered In Vitro Multi‐Cell Type Ventricle Model Generates Long‐Term Pulsatile Flow and Modulates Cardiac Output in Response to Cardioactive Drugs

**DOI:** 10.1002/adhm.202403897

**Published:** 2025-02-13

**Authors:** Christoph Kuckelkorn, Ebru Aksoy, Natalija Stojanovic, Laila Oulahyane, Mira Ritter, Kurt Pfannkuche, Horst Fischer

**Affiliations:** ^1^ Department of Dental Materials and Biomaterials Research RWTH Aachen University Hospital Pauwelsstrasse 30 52074 Aachen Germany; ^2^ Center for Physiology and Pathophysiology Institute for Neurophysiology University and University Hospital of Cologne Robert Koch Str. 39 50931 Cologne Germany; ^3^ Center for Molecular Medicine Cologne (CMMC) 50931 Cologne Germany; ^4^ Marga‐and‐Walter‐Boll‐Laboratory for Cardiac Tissue Engineering 50931 Cologne Germany

**Keywords:** cardiomyocyte, engineered ventricle, in vitro model, pulsatile flow

## Abstract

Cardiac in vitro models serve as promising platforms for physiological and pathological studies, drug testing, and regenerative medicine. This study hypothesizes that immobilizing cardiomyocytes derived from human induced pluripotent stem cells (iPSC‐CMs) on a biofunctionalized, hemispherical membrane can generate pulsatile flow through synchronized contractions, thus offering as an in vitro left ventricle model. To test this, a ventricle using a polydimethylsiloxane (PDMS) membrane coated with polydopamine and laminin 511 E8 fragments is engineered. Human iPSC‐CMs are cultured on these membranes, alone or in co‐culture with cardiac fibroblasts or endothelial cells, for 28 and 14 days, respectively, in a newly developed bioreactor. Flow measurements track beating and flow generation, while drug response, cardiac gene expression, and cell morphology are analyzed. The engineered ventricles maintain continuous beating and flow, achieving a theoretical cardiac output of up to 4 µL min^−1^ over 28 days, indicating stable cell adhesion and synchronized contraction. Cardiomyocytes respond to cardioactive drugs (carbachol, isoproterenol) and show expected changes in heart rate and cardiac output. In conclusion, the results demonstrate that the proposed engineered ventricle can serve as an in vitro left ventricle model by supporting cardiomyocyte culture and differentiation, generating long‐term stable flow, and responding physiologically to cardioactive drugs.

## Graphical abstract

This study presents an innovative in vitro model of a human left ventricle using biofunctionalized PDMS membranes and human iPSC‐derived cardiomyocytes. The engineered ventricle demonstrates stable, long‐term pulsatile flow, and physiological responses to cardioactive drugs. It enhances cardiomyocytes maintenance and differentiation, making it a valuable tool for drug testing and advancing cardiac research applications.

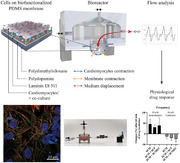

## 1 Introduction

Cardiovascular diseases continue to be the leading cause of death worldwide.^[^ ^1^ ^]^ To understand physiological correlations and diseases as well as to develop appropriate therapies, in vitro models followed by animal experiments are used.^[^ ^2^, ^3^, ^4^, ^5^ ^]^ Disadvantages of animal experiments include the inability to reconstruct human‐specific aspects of heart development, histogenesis, and human physiology. Aside from ethical considerations, they are also associated with high costs and significant time expenditure.^[^ ^3^, ^6^, ^7^, ^8^ ^]^ On the other hand, cardiac in vitro models for investigating cardiovascular diseases significantly lag behind the capabilities of other organ models such as kidney, intestine, or brain.^[^ ^9^ ^]^ Although human induced pluripotent stem cells (hiPSCs) can be used to generate almost all required cell types in sufficient numbers for in vitro models,^[^ ^10^, ^11^, ^12^ ^]^ current in vitro models for pharmaceutical screens are usually limited to one or very few cell types or lack the ability to mimic the blood‐flow generating pumping function of the heart through appropriate geometry and synchronous contraction.^[^ ^13^, ^14^ ^]^

Research conducted by Tanaka et al. demonstrated in 2007 that applying neonatal rat cardiomyocytes to a fibronectin coated polymeric sphere could induce sphere contraction and generate pulsatile flow.^[^ ^15^ ^]^ However, the cardiomyocyte layer experienced detachment and dedifferentiation within six days. This highlights the critical need to achieve long‐term stable biofunctionalization of the polymer and the usage of human iPSC‐derived cardiomyocytes, which do not dedifferentiate and maintain their contractile properties over time.^[^ ^16^, ^17^ ^]^ Another research group utilized bioprinting techniques to fabricate structures resembling ventricles. However, this approach failed to achieve macroscale contractile function due to either insufficient cell concentrations or a lack of prolonged in vitro culture.^[^ ^14^, ^18^ ^]^ Lee et al. successfully fabricated a contracting ventricle utilizing the FRESH technique, achieving a cell density approaching physiological levels.^[^ ^19^ ^]^ MacQueen et al. utilized an electrospun scaffold, that they seeded with hiPSC‐CMs and reached contractile work 10^4^–10^8^ times smaller than the corresponding values for human ventricles.^[^ ^20^ ^]^ In another study, the cell density could be adequately increased through in situ proliferation of iPSCs before their cardiac differentiation, thereby achieving synchronized contraction.^[^ ^21^ ^]^ However, this approach is limited in that iPSCs can only be differentiated into a single cell type, precluding the possibility of co‐culture. This restriction hampers the creation of a more physiological environment, essential for mimicking the complex interactions within the heart. In vitro models that provide a more physiological cellular environment by incorporating multiple cell types include spheroids, organoids and engineered heart tissue.^[^ ^13^, ^22^ ^]^ These models are proficient in generating force and exhibit predictable responses to pharmacological agents.^[^ ^23^, ^24^ ^]^ Unfortunately, such models are unable to generate a pulsatile flow and therefore they lack the main function of the human heart.^[^ ^13^, ^14^ ^]^

Polydimethylsiloxane is a silicone‐based synthetic polymer often used for in vitro models,^[^ ^25^, ^26^, ^27^ ^]^ due to its low costs, ease of handling, good mechanical stability, and tunable stiffness. In particular, the ability to tune its stiffness allows for creation of a substrate which that can be customized to mimic native tissue^[^ ^28^ ^]^ like the myocardium (4–330 kPa ^[^ ^29^ ^]^) instead of traditional tissue culture plates (3–3.5 GPa ^[^ ^30^ ^]^). Moreover, PDMS is characterized by its cytocompatibility, optical transparency, and gas permeability.^[^ ^28^, ^31^ ^]^ A major challenge, however, is its hydrophobic characteristics which arise as a result of the bond of two methyl groups on each silicon atom.^[^ ^32^ ^]^ As a result, cells cannot easily adhere to PDMS, making polymer modification essential in order to create a more reactive interface. Techniques to enhance hydrophilicity and modify PDMS for improved cell adhesion include UV light treatment, acid treatment, plasma treatment, classic protein deposition, and polydopamine (PDA) treatment, as well as the use of organosilanes, like 3MOBS, and the combination of the aforementioned approaches.^[^ ^25^, ^31^, ^33^, ^34^ ^]^ For protein deposition, laminin E8 511, which represents the minimal fragment size capable of integrin‐binding activity, has been recognized as a significant regulator of cardiomyocyte maturation for the differentiation of iPSCs.^[^ ^35^ ^]^ For the culture of iPSCs it has even been reported that laminin E8 fragments promote greater adhesion than full length laminin, Matrigel or fibronectin.^[^ ^36^ ^]^

When addressing the challenges associated with selecting an appropriate substrate and its functionalization for an in vitro model, it is equally important to recognize the difficulties of creating a physiologically relevant model at the cellular level. This involves not only the incorporation of cardiomyocytes but also the integration of various cardiac cell types, such as fibroblasts and endothelial cells, which are crucial for maintaining heart structure and function. In vivo, cardiac fibroblasts (CFs) are known to synthesize, degrade, and remodel the extracellular matrix (ECM), as well as respond to a broad spectrum of chemical signals involved in the paracrine and autocrine regulation of cardiac function.^[^ ^37^, ^38^ ^]^ Endothelial cells (ECs) also participate in regulating and sustaining cardiac function both at the endocardium and within myocardial capillaries, where they interact directly with neighboring cardiomyocytes. ECs are capable of secreting growth factors to preserve the phenotype and survival of cardiomyocytes and express and release a range of auto‐ and paracrine agents, which directly affect cardiac metabolism, growth, contractile performance, and rhythmicity.^[^ ^39^, ^40^ ^]^

In our study, we hypothesize that by immobilizing cardiomyocytes differentiated from human induced pluripotent stem cells (iPSCs) on a hemispherical tailored biofunctionalized polydimethylsiloxane (PDMS) membrane, we can achieve a pulsatile flow through the synchronous contraction of the immobilized cells without the need for a 3D printing or electrospinning system. Furthermore, this engineered ventricle is anticipated to support co‐cultures and exhibit physiological responses to pharmacological agents. An important innovation of the proposed model is the ability to directly measure and quantify the cell responses by assessing cardiac output in a multi‐cell type environment.

**Figure 1:**
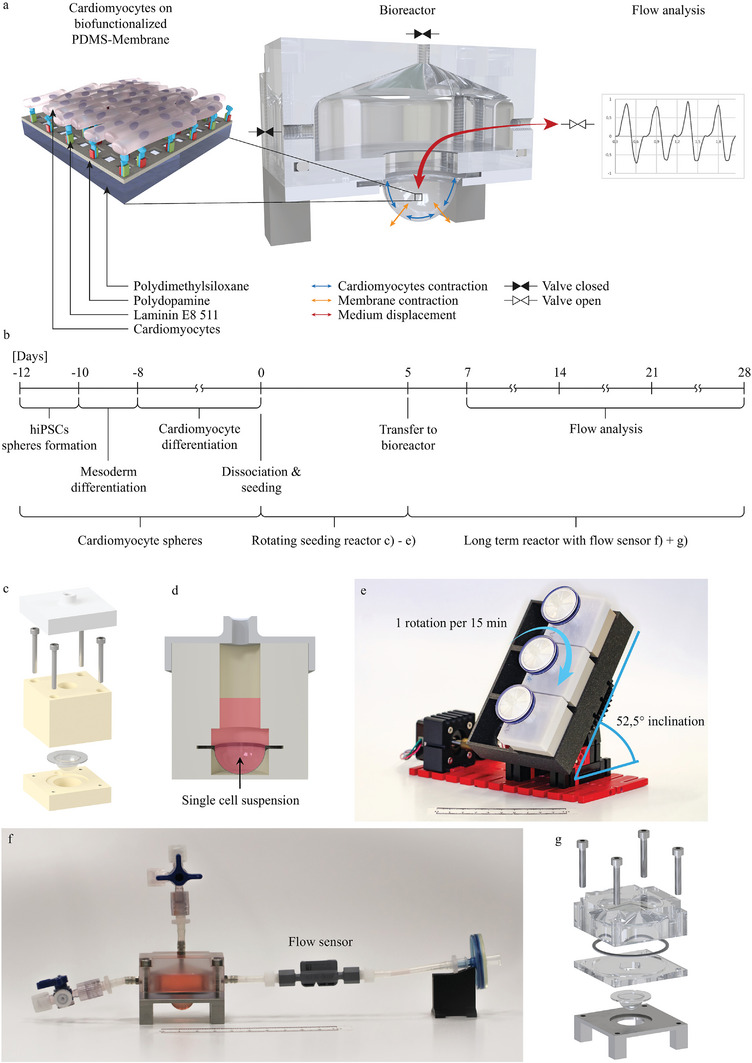
a) Schematic depiction of the bioreactor housing the biofunctionalized hemispherical PDMS membrane. Contraction of the immobilized cardiomyocytes on the membrane induces contraction of the membrane itself, resulting in volume displacement that can be quantified using a flow sensor. b) Time scale of the experiment to measure the contraction dynamics of the engineered ventricles. c) CAD exploded view of the seeding reactor, highlighting its individual components. d) CAD cross section of the seeding reactor with the area where the single cell suspension is added marked. e) Image of the rotating seeding reactor showing the 52.5° inclination angle and the rotation axis of the apparatus, which ensures uniform distribution of cells over the entire membrane. f) Photograph of the long‐term cultivation bioreactor, optimized for continuous flow measurements. g) CAD exploded view of the long‐term cultivation bioreactor, providing a detailed visualization of its structural elements.

Briefly, to confirm our hypothesis, we compare common functionalization methods of PDMS for cardiomyocytes immobilization to determine the most effective technique. Besides different pre‐treatments for the oxidation of PDMS, we evaluate PDA and trimethoxy‐[2‐(7‐oxabicyclo[4,1,0]hept‐3‐yl)ethyl]‐silane (3MOBS) as crosslinker for adhesion proteins. To achieve a homogeneous and confluent distribution of cells within the hemispherical membrane, we engineer a novel custom‐made bioreactor. This device uses a tilted axis of rotation to evenly distribute the cells across the membrane surface. Once a confluent layer of cells is established, the hemispherical membrane is transferred to another specially designed bioreactor. This second bioreactor facilitates long‐term cultivation while allowing measurement of the flow/cardiac output generated by the contracting cardiomyocytes over a period of 28 days (**Figure** **1**a). We conduct experiments on both monocultures of cardiomyocytes and co‐cultures with either cardiac fibroblasts or cardiac endothelial cells, to dissect how each cell type can influence the engineered ventricle. In addition to analyzing the beating and flow characteristics of our in vitro model, we also characterize the cells by flow cytometry, immunofluorescence staining, qPCR, and evaluate their response to cardioactive drugs, providing a comprehensive assessment of the functionality of the model and its potential application in cardiac research.

## 2 Experimental Section

### 2.1 Human Pluripotent Stem Cell Culture

The human induced pluripotent stem cell line NP0040‐8 (provided by Dr. Tomo Saric, University of Cologne, Medical Faculty, Institute for Neurophysiology) was cultured in 6 cm petri dishes that had been pre‐coated with Matrigel matrix (734–1440, Corning, Darmstadt, Germany) at a seeding density of 10 µg cm^−2^ growth area. The culture medium used was E8, which consisted of DMEM/F12 (1:1) with Glutamax (31 331‐028, Thermo Fisher Scientific, Waltham, USA) supplemented with: 100 µg mL^−1^ L‐ascorbic acid phosphate magnesium n‐hydrate (013–12 061, Wako Chemicals, Osaka, Japan), 20 µg mL^−1^ insulin (“Humalog 100I.E.”, Lilly Deutschland, Bad Homburg, Germany), 5 µg mL^−1^ transferrin (Sigma–Aldrich, #T3705), 14 ng mL^−1^ sodium selenite (Sigma–Aldrich, #S5261), 100 ng mL^−1^ heparin sodium salt (H3149, Sigma–Aldrich, Darmstadt, Germany), 100 ng mL^−1^ basic fibroblast growth factor 2 (100‐18B, Peprotech, Hamburg, Germany), and 2 ng mL^−1^ transforming growth factor β (100–21, Peprotech, Hamburg, Germany). These cells were maintained in a humidified incubator at 37 °C with 5% CO_2_. To ensure healthy growth, the hiPSCs were sub‐cultured every 3–4 days when the cell culture reached 70–80% confluency. This process involved removing the medium, washing the culture dish with 1 mL DPBS (−/−), and adding 1 mL of ReLeSR (5872, Stemcell Technologies, Cologne, Germany) to 6 cm petri dishes. The mixture was incubated for 5 min at room temperature, after which the ReLeSR was removed. The plate was then incubated for an additional 5 min in a humidified incubator at 37 °C with 5% CO_2_. Subsequently, 50–200 µL of the hiPSC cluster suspension was transferred into fresh wells of Matrigel‐coated 6 cm petri dishes, and 5 mL of E8 medium was added supplemented with 10 µM of Rho Kinase (ROCK) inhibitor (Y27 632, Adooq, #A11 001‐5). The plate was incubated in a humidified incubator at 37 °C with 5% CO_2_. A complete medium change was performed every 24 h, transitioning to E8 medium without the ROCK inhibitor.

### 2.2 Generation of hiPSC‐CM in 3D Culture

For differentiation of hiPSCs into CMs, a hiPSCs single‐cell suspension was used. When the cell culture reached 70–80% confluency, the single‐cell suspension of hiPSCs was prepared. The culture was first washed with DPBS (−/−), then dissociated with ReLeSR. Subsequently, the cell suspension was filtered through a 40 µm cell strainer (542 040, Greiner Bio‐One, Frickenhausen, Germany) centrifuged at 120 g, and resuspended in 1 mL of E8 medium supplemented with 10 µM ROCK inhibitor. The cell count on a hemocytometer with Trypan Blue, and 0.3 × 10^−6^ hiPSCs per mL was added to 6 cm petri dishes containing 5 mL of E8 medium supplemented with 10 µM ROCK inhibitor. This marked the starting day of cardiac sphere formation, referred to day −12. The hiPSCs were cultured on a 60‐rpm linear shaker at 37 °C with 5% CO_2_ for 48 h. On day −10, mesodermal induction was initiated by completely replacing the medium with RPMI 1640 (61 870‐010, Thermo Fisher Scientific, Waltham, USA) containing 1× B27 supplement without insulin (175 044, Thermo Fisher Science, Waltham, USA) and 12 µM CHIR99 021 (C‐6556, LC Laboratories, Woburn, USA) on a 60‐rpm linear shaker at 37 °C with 5% CO_2_ for 24 h. On day −9, a complete medium change was performed using RPMI 1640 supplemented with 1× B27 without insulin and 100 µg mL^−1^ ascorbate, without CHIR99 021 supplementation. At day −8, 72 h after the initiation of differentiation, half of the medium was changed to RPMI 1640 with 1× B27 supplement without insulin, 100 µg mL^−1^ ascorbate, and final concentrations of 10 µM IWP2 (3 533 110, Tocris Bioscience, Bristol, UK) and 10 µM XAV939 (X3004, Sigma–Aldrich, Darmstadt, Germany) on a 60‐rpm linear shaker at 37 °C with 5% CO_2_ for 48 h. Starting from day −6, the culture medium was changed to RPMI 1640 with 1× B27 supplement without insulin and with 100 µg mL^−1^ ascorbate, and it was replaced every 3 days. A visual representation of the process is shown in Figure 1b.

### 2.3 Dissociation of CM Spheres

At day 0 cardiomyocyte spheres were dissociated with 0.25% Trypsin + EDTA (25 200 056, ThermoFisher Scientific, Waltham, USA), 0.1mg mL^−1^ collagenase type II (17 101‐015, ThermoFisher Scientific, Waltham, USA) and 0.15 mg mL^−1^ DNase I (11 284 932 001, Sigma–Aldrich, Darmstadt, Germany) to cut released DNA from dead cells. During the dissociation (40 min), the cells were incubated at 37 °C and pipetted up and down seven times every 10 min with a 1000 µL pipette tip. Trypsin reaction was stopped with RPMI 1640 Medium containing 10% FBS (26 140 079, ThermoFisher Scientific, Waltham, USA).

### 2.4 Cultivation of Cardiac Fibroblasts

Human cardiac fibroblasts (hCFs) isolated from the ventricles of the adult heart were purchased (C‐12 375, PromoCell, Heidelberg, Germany) and cultivated in DMEM/F‐12 medium (11 320 033, ThermoFisher Scientific, Waltham, USA) supplemented with 5% ELAREM Perform‐FD PLUS (PE30 811, PL BioScience GmbH, Aachen, Germany), 1% penicillin/streptomycin, 1 ng mL^−1^ fibroblast growth factor (100‐18b, PeproTech, Waltham, USA), and 5 µg mL^−1^ insulin (I9278, Simga–Aldrich, Darmstadt, Germany). When the hCFs reached 70–90% confluency they were passaged using trypsin/EDTA (P10‐023 100, PanBiotech, Aidenbach, Germany) at a plating density of 7000 cells cm^−2^.

### 2.5 Cultivation of Cardiac Endothelial Cells

Human cardiac microvascular endothelial cells (hCMEC) isolated from heart ventricles were purchased (C‐12 285, PromoCell, Heidelberg, Germany) and cultivated in T75 cell culture flasks previously coated with 2% (w/v) gelatin. The growth medium was endothelial cell basal medium (C‐22 111, PromoCell, Heidelberg, Germany) supplemented with 1% penicillin/streptomycin and modified to a final FCS concentration of 5%. When the HCMECs reached >70% confluency they were passaged using trypsin/EDTA (P10‐023 100, PanBiotech, Aidenbach, Germany) at a plating density of 15.000 cells cm^−2^.

### 2.6 Donor information

As differences in cellular phenotype, behavior and composition occur in the male and female hearts,^[^ ^41^ ^]^ we used primary cells of the same gender and ethnicity without any known diseases in order to receive a consistent in vitro model. The donor information is presented in **Table** **1**.

**Table 1:** Donor information of primary cells used.

Cells	Lot/ID	Gender	Age	Ethnicity
hiPSC	NP0040‐8	Male	35–39	Caucasian
hCF	479Z018.2	Male	43	Caucasian
hCMEC	488Z023.3	Male	36	Caucasian

### 2.7 Cardiomyocyte attachment experiment

Single cell suspension of CMs was pipetted on the functionalized PDMS samples in a 24 well plate at a density of 500 000 CMs cm^−2^. Culture medium (RPMI 1640, 1x B27 supplement minus insulin, 100 µg mL^−1^ ascorbate and 1% penicillin/streptomycin) was exchanged every 2–3 days. Brightfield microscopy images were taken with Primovert Microscope and Axiocam 208 color (Carl Zeiss Microscopy Deutschland, Oberkochen, Germany) at the following time points: 2 h, 48 h, and 8 days post seeding. Videos of beating CMs were captured with the same microscope at days 7 and 14 post seeding. Optical flow of the captured CM beating was calculated using Maia software (QuoData GmbH, Dresden, Germany; Algorithm: Gunnar Farnbeäck, Frame offset 6, Iterations 3, Pyramid scale 0.5, Neighborhood 5, Number of pyramids 3, Gauss StdDev. 1.2, Window size 15, Flags 0). As the Maia software only calculates the optical flow, with positive flow values an integration of every beat was performed using a MATLAB (MathWorks, Natick, USA) script. To circumvent the issue of only positive flow values, a reversal of sign was implemented during the integration. This specific point (reversal of sign) was chosen such that the integration's boundary conditions stipulated that the computed displacement, after a beat, return to the initial point of origin.

### 2.8 Immunofluorescence

Images were taken using an Axio Imager 2 microscope (Carl Zeiss Microscopy Deutschland, Jena, Germany) and an inverted Zeiss LSM 980 with Airyscan 2 laser scanning confocal microscope (CLSM; LSM 980, Carl Zeiss Microscopy, Jena, Germany) equipped with a LD LCI Plan‐Apochromat 25x (N.A. 0.8) multi‐immersion objective and Zen Blue 3.9 software (Carl Zeiss Microscopy Deutschland, Jena, Germany). With the Zeiss LSM 980, images were acquired as z‐stacks either in LSM Plus mode or super‐resolution (SR) mode using the Airyscan 2 detector and followed by the corresponding deconvolution method. Samples were prepared by fixing them in 4% formaldehyde for 10 min. This step was followed by cell permeabilization with the solution of 0.1% of TritonX‐100 in 1xPBS for 15 min and incubation in 3% bovine serum albumin of blocking solution for 30 min. Subsequently, the samples were incubated overnight at 4 °C with primary antibody. Next, incubation with secondary antibody was performed for 60 min at room temperature, and the nuclei were counterstained with 4′,6‐diamidino‐2‐phenylindole (DAPI) (1:2000, Thermo Fisher Scientific, Waltham, USA). List of the primary and secondary antibodies used can be found in Table S1 (Supporting Information).

### 2.9 Contact Angle Measurements

To quantify the hydrophilicity of the functionalized PDMS, contact angle measurements were performed by pipetting 2.5 µL deionized water on the surface. Pictures were taken immediately after placing the droplets using a COE‐032‐M‐POE‐040‐IR‐C camera and TC23 007 objective connected with OECS software, all from OPTO Engineering (Grünwald, Germany). The contact angles were then calculated with the ImageJ software using the contact angle plug‐in. Three samples from each group were tested with three different droplets on every sample resulting in nine measurements per group. Measurements were taken at day 1 and day 21. For measurements at day 21, new samples were used so that there was no need to dry beforehand, and they could be stored in PBS at 4 °C.

### 2.10 PDMS Sample Preparation

Each PDMS sample was prepared by mixing elastomer and curing agent at a ratio of 25:1 w/w. Afterward 40 µL of PDMS was pipetted on a 12 mm glass coverslip. The PDMS was then cured for 48 h at 60 °C. Next, samples were sterilized by autoclaving at 120 °C. In cases where a UV treatment was performed, the samples were placed for 60 min at 3 cm distance from the light source (G15T8, Sonkyodenki, Eschborn, Wavelength: 253.9 nm, UV output: 4.9 W, Intensity at 3 cm distance: 4460 µW cm^−2^). For chemical oxidation, samples were submerged for 3 h in a 1:1 mixture of hydrochloric acid 37% (1.00314.100, Sigma‐Aldrich, Darmstadt, Germany) and hydrogen peroxide 30% (1.08597.1000, Sigma–Aldrich, Darmstadt, Germany).

### 2.11 Polydopamine Functionalization

Dopamine hydrochloride (H8502‐25G, Sigma–Aldrich, Darmstadt, Germany) was dissolved in a 10 mM TRIS buffer (252 859, Sigma Aldrich, Darmstadt, Germany) to a final concentration of 0.01% w/v. Previously the pH of the TRIS buffer had been adjusted to 8.5 using HCl. PDMS samples were submerged into the dopamine solution for 24 h at room temperature.

### 2.12 3MOBS Functionalization

When using solvent deposition of 3MOBS, first a buffer made of isopropanol (20 842 330, VWR Chemicals, Darmstadt, Germany) and di‐water was mixed at ratio of 19:1. This buffer was set to pH 5 using acetic acid. Afterward, 3MOBS (413 321, Sigma–Aldrich, Darmstadt, Germany) was added to the buffer to a final concentration of 2% v/v. PDMS samples were submerged in the 3MOBS solution for 3 h at room temperature. For vapor deposition, the PDMS samples were placed in a round bottom flask with a vacuum valve. Pressure inside the flask was lowered to 0.5–1 mbar and 100 µL of pure 3MOBS was injected via a silicone cap with the help of a needle. Finally, the flask with the samples was placed in an oven for 1 h at 120 °C.

### 2.13 Protein Deposition

For the cardiomyocyte experiments, laminin E8 511 fragments were diluted in PBS to a final concentration of 2 µg mL^−1^. Functionalized PMDS samples were submerged in the Laminin E8 solution for 24 h at 37 °C. For contact angle measurements, we used collagen 1 (5005, Advanced BioMatrix, Carlsbad, USA) at a concentration of 100 µg mL^−1^ for 1 h at 37 °C instead of laminin E8. This was done in order to be able to compare the results with results from Chuah et al., who did important research in characterizing PDMS, dopamine and collagen 1 interaction for immobilization of human mesenchymal stromal cells.^[^ ^31^ ^]^ Abbreviations listed in **Table** **2** are used in the following text and figures.

**Table 2:** Abbreviations used in the following to make illustrations and text clearer.

Abbreviation	Meaning/full name
PDMS	Untreated Polydimethylsiloxane
UV	Pretreatment with UV light
PDA	Polydopamine
AC	Pretreatment with acid solution
3MOBS	Trimethoxy[2‐(7‐oxabicyclo[4,1,0]hept‐3‐yl)ethyl]silane
Protein	Collagen I for contact angle measurements, Laminin E8 511 for cardiomyocyte experiments

### 2.14 Cell Seeding on Hemispherical Membrane

PDMS was prepared according to the previously outlined procedure and cast into an aluminum mold comprising upper and lower segment. The resulting membrane exhibited a thickness of mean = 115.7 µm with a standard deviation = 18.7 µm (Figure S4, Supporting Information). Following the curing process, the hemispherical membranes were autoclaved and subjected to functionalization with PDA and Laminin E8 511. A custom‐designed rotating seeding bioreactor was fabricated for the purpose of seeding single‐cell suspensions onto the hemispheres (Figure 1c,d). The membrane was positioned inside the reactor and placed within an apparatus, that tilts the reactor 52.5° while rotating at a rate of 1 rotation per 15 min (Figure 1e). After cell dissociation as described previously, the single‐cell suspension was transferred into the rotational seeding reactor (Figure 1f,g) at concentrations delineated in **Table** **3**.

**Table 3:** Cell concentration seeded on the hemispherical PDMS membranes at day “0”.

Culture Type	Cardiomyocytes cm^−2^	Endothelial cells cm^−2^	Fibroblasts cm^−2^
Pure Cardiomyocyte	750 000	–	–
Co‐culture	750 000	50 000	–
Co‐culture	750 000	–	10 000

The entire setup was placed inside an incubator (5% CO_2_ and 37 °C). The culture medium inside the seeding reactor was replaced daily. For cultivation of pure cardiomyocytes RPMI 1640 Medium, supplemented with 1x B27 supplement minus insulin, 100 µg m^−2^ ascorbate and 1% Penicillin/streptomycin was used. In the case of co‐culture experiments, a mixture comprising 50% of the aforementioned RPMI 1640 Medium with supplements and 50% EBM‐2 Medium (C‐22 111, PromoCell, Heidelberg, Germany) was used.

### 2.15 Long Term Cultivation/Flow Analysis Reactor

To measure the displacement of the medium due to cardiomyocyte contraction, a custom bioreactor was designed (Figure 1). The cell‐laden membrane was transferred from the rotating seeding reactor into the long‐term cultivation reactor on day 5. The reactor features three connection ports, two on the lateral sides and one positioned at the apex of the medium chamber, facilitating medium filling and expulsion of potential gas bubbles. This design consideration is crucial to preventing distortion in volume flow measurements as gas bubbles, being compressible, may behave differently from the incompressible medium. Flow data was captured using an SLF3S‐0600F flow sensor (Sensirion, Stäfa, Switzerland), connected to a lateral port of the reactor. The USB Sensor Viewer Software (Sensirion, Stäfa, Switzerland) was employed to export the flow data into comma‐separated values (.csv) format. Experiments were conducted with *n* = 5 engineered ventricles at pure cardiomyocyte culture and *n* = 3 for co‐culture measurements. To test the response to sympathetic and parasympathetic stimuli, the engineered ventricles were exposed to the beta‐adrenoceptor agonist isoproterenol (10 µM, I5627‐5G, Sigma–Aldrich, Darmstadt, Germany) and the cholinergic receptor antagonist carbachol (10 µM, C4382‐1G, Sigma‐Aldrich, Darmstadt, Germany). A control flow measurement was performed before drug incubation, and the reaction was measured after 60 min of drug incubation. For better comparison, the data were normalized. To assess the response to cardioactive drugs, *n* = 4 ventricles were tested per group.

### 2.16 Flow Cytometry Analysis

For flow cytometry analysis, cells were dissociated as previously described. Antibody labeling was performed similarly to the immunofluorescence staining, with the exception that the single‐cell solution was centrifuged at 300 g for 3 min after each step to allow the removal of the supernatant without disturbing the cell pellet. Measurements were conducted using a FACSCanto II Clinical Flow Cytometry System (Becton, Dickinson and Company, New Jersey, USA).

### 2.17 qPCR Analysis

Depending on the sample, cells were dissociated as previously described on days 0, 14, and 28 (**Table** **4** For co‐culture experiments, non‐cardiomyocytes were depleted using magnetic activated cell sorting (MACS) (130‐110‐188, Miltenyi Biotec, Bergisch Gladbach, Germany) following the manufacturer's instructions. Subsequently, the cells were lysed, and RNA was isolated using the RNeasy Mini Kit (Qiagen, Hilden, Germany) in accordance with the manufacturer's protocol. The isolated RNA was then used for cDNA synthesis with the RevertAid First Strand cDNA Synthesis Kit (Thermo Fisher Scientific, Waltham, USA) according to the kit's guidelines. After synthesis, cDNA concentration was measured using Quantifluor ONE dsDNA dye and Quantus Fluorometer (Promega, Madison, USA). After measuring, cDNA concentration was set to 0.1 ng µL^−1^ and used further in qPCR. QuantiNova SYBR Green PCR Kit (Qiagen, Hilden, Germany) was used to perform qPCR, and primers were designed with Primer‐BLAST^[^ ^42^ ^]^ and ordered from Eurofins Genomics (Eurofins Genomics, Luxembourg). Reaction mixes following the manufacturer's recommendation as well as the primer sequences used in qPCR are provided in Tables S2 and S3 (Supporting Information). Genes of interest were normalized to the housekeeping gene Glyceraldehyde 3‐phosphate dehydrogenase (GAPDH). ΔΔCt method was used to calculate the fold change in gene expression change.

**Table 4:** Time points at which RNA was isolated, depending on the sample type.

Sample/Day of mRNA Isolation	Day 0 (dissociated cells)	Day 14	Day 28
hCM on tissue culture plate	Yes	No	Yes
hCM cardiac spheres	Yes	No	Yes
hCM engineered ventricle	Yes	Yes	Yes
hCM + hCF engineered ventricle	Yes	Yes	No
hCM + hCMEC engineered ventricle	Yes	Yes	No

### 2.18 Statistical Analysis

Statistical analysis was performed using GraphPad PRISM 10 (GraphPad Software, San Diego, USA). *t*‐test, ANOVA and two‐way ANOVA tests were performed to study significant differences within groups, followed by Tukey's post‐hoc test for comparison between groups. Significance levels of ^*^ *p* < 0.05, ^**^ *p* < 0.01, ^***^ *p* < 0.001, and ^****^ *p* < 0.0001 were defined.

## 3 Results

### 3.1 Biofunctionalization of PDMS for Adherence of Cardiomyocytes

**Figure 2:**
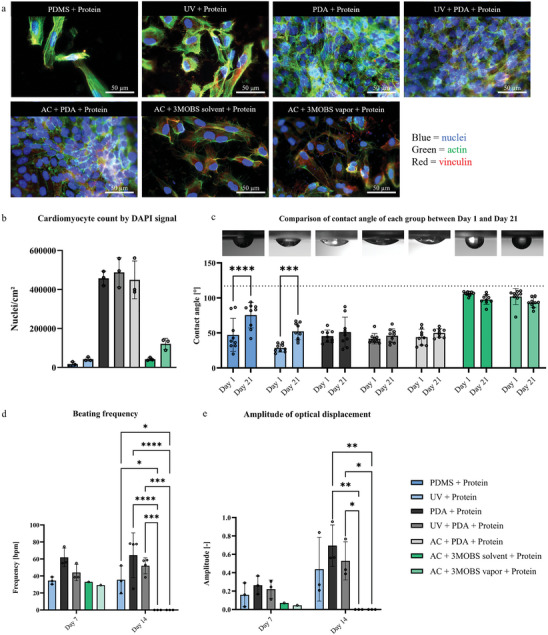
a) Immunofluorescence micrographs showcasing cardiomyocytes adhered to 2D PDMS samples following various functionalization techniques, including crosslinking of proteins with polydopamine (PDA) and 3MOBS silane, after a 14‐day period (scale bar = 50 µm). b) Quantification of adherent cardiomyocytes count by nuclei staining on different functionalized PDMS (the significance levels are shown in Table S4, Supporting Information, for the sake of clarity). c) Contact angle measurements of PDMS following various functionalization methodologies (significance is only displayed within each group between day 1 and 21. Comprehensive comparisons across all groups can be referenced in Table S5, Supporting Information). Assessment of the frequency d) and amplitude of optical displacement e) corresponding to cardiomyocyte cultures beating patterns (only groups are displayed, where cardiomyocytes were beating synchronized once during the experiment). The statistical analyses were carried out using one‐way ANOVA (b) and two‐way ANOVA (c–e). *n* = 3 experiments for b), *n* = 9 technical replicates for c), *n* = 4 experiments for d,e). Significance levels of ^*^ *p* < 0.05, ^**^ *p* < 0.01, ^***^ *p* < 0.001, and ^****^ *p* < 0.0001 were defined.

Immunofluorescence staining for actin (green) and vinculin (red) at day 14 proved high cell densities in all dopamine treated PDMS groups (**Figure** **2**a). A dense network of expressed actin fibers can be seen in these groups. Vinculin expression was visible at the end of the actin fibers, indicating focal adhesion. Apart from that, the actin fibers showed no uniform orientation of the cardiomyocytes. The 3MOBS treated groups as well as the classical deposition and UV‐treatment group showed a lower number of cells (Figure 2b) but no great differences in morphology, actin cytoskeleton or vinculin expression. Brightfield microscopy images of the cardiomyocytes after different time periods can be seen in Figure S1 (Supporting Information).

Contact angle measurements taken on days 1 and 21 show significant increases over the three‐week period in the untreated PDMS and the UV‐treated PDMS group (both + adhesion proteins), indicating hydrolytically unstable behavior (Figure 2c). No other groups revealed significant differences between days 1 and 21 and therefore exhibited hydrolytically stable behavior. Among these groups, dopamine treated PDMS demonstrated the lowest contact angles, with a mean ranging between 46° and 51° on day 21.

The start of the cardiomyocytes’ beating was between days 2 and 4. Synchronously beating cardiomyocytes were analyzed with regard to the amplitude of optical displacement and frequency of beating at days 7 and 14 (Figure 2d). Amplitude of beating increased from day 7 to 14 in the UV‐ and PDA‐treated groups. 3MOBS groups only showed synchronous beating at day 7. At day 14, the three remaining beating groups (UV treatment, only dopamine, and combination of UV and dopamine) showed no significant differences in the beating amplitude. However, significant differences were observed in the frequency of beating. The group of combined UV and dopamine treatment showed a mean contraction frequency of 64.5 bpm, followed by the only dopamine treatment showing 52.0 and 35.5 bpm for the UV treatment group. Analysis of samples functionalized with acid pretreatment in combination with dopamine was not possible due to the lack of transparency of etched PMDS.

The PDMS substrate, prepared at a 25:1 ratio, was further analyzed using a rotational rheometer. The elastic shear modulus (𝐺′) was evaluated as a function of frequency and strain amplitude, as shown in Figure S2 (Supporting Information). Within the measured ranges, the shear modulus showed minimal variation, maintaining values between 12.4 and 14.7 kPa.

### 3.2 Engineered Ventricle Model Using a Monoculture of Cardiomyocytes

**Figure 3:**
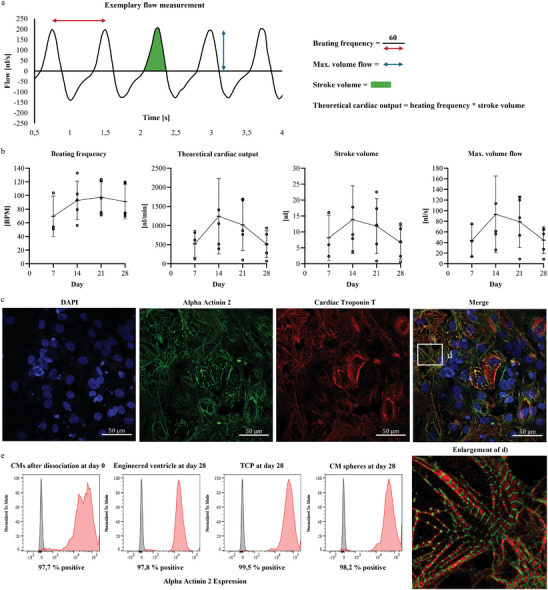
a) Exemplary flow measurement of an engineered ventricle with representation of the analyzed parameters b) Analysis of beating frequency, theoretical cardiac output, stroke volume and the max. volume flow over the period of 28 days of engineered ventricle seeded only with cardiomyocytes. No statistical significance levels were found by one‐way ANOVA between the different days in each analyzed parameter. Data of *n* = 5 engineered ventricles were used for statistical analysis. Significance levels of ^*^ *p* < 0.05, ^**^ *p* < 0.01, ^***^ *p* < 0.001, and ^****^ *p* < 0.0001 were defined. c) Immunofluorescence staining of alpha actinin 2 (green) and cardiac troponin T (red) and nuclei (blue) of cardiomyocytes from the engineered ventricle. Scale bar = 50 µm. d) Characteristic section of merged image which is displayed enlarged for a better visibility. e) Flow cytometry analysis of cardiomyocytes after dissociation (day 0), the engineered ventricle, TCP (tissue culture plate), and CM sphere cultures at day 28 for alpha‐actinin 2 expression.

An sample flow measurement of an engineered ventricle seeded with pure cardiomyocytes is shown in **Figure** **3**a, illustrating the parameters analyzed: beat frequency, theoretical cardiac output (calculated as the product of frequency and stroke volume), displaced volume per beat (stroke volume), and maximum volumetric flow rate. The ventricles showed long‐term stable beating characteristics, that persisted until the end of the measurements on day 28 (Figure 3b). In addition, brightfield microscopy and calcium transient fluorescence imaging were performed to confirm the synchronous contraction of the cardiomyocytes (Supplementary Videos). The mean beating frequency ranged from 69.6 bpm on day 7 to 97.5 bpm on day 21. The theoretical cardiac output, stroke volume, and maximum volume flow tended to increase until day 14 and then decrease though day 28. However, these differences were not statistically significant. The mean theoretical cardiac output ranges from 0.5 µL min^−1^ on day 28 to 1.2 µL min^−1^ on day 14. The stroke volume ranges from 6.7 to 13.8 nL. Immunofluorescence images revealed a confluent cardiomyocyte layer with well‐developed sarcomere structures, as indicated by alpha‐actinin 2 and cardiac troponin staining (Figure 3c,d). The sarcomere length was measured to 2.03 µm ± 0.16 µm. Additionally, flow cytometry showed that the number of cells expressing alpha‐actinin 2 was greater than 97.7% in all examined groups, which included the single cell suspension during seeding on day 0 and cells from the engineered ventricle, cells on TCP, and cells in cardiac sphere cultivation on day 28 (Figure 3e).

### 3.3 Gene Expression Profiles of Cardiomyocytes from Engineered Ventricle

**Figure 4:**
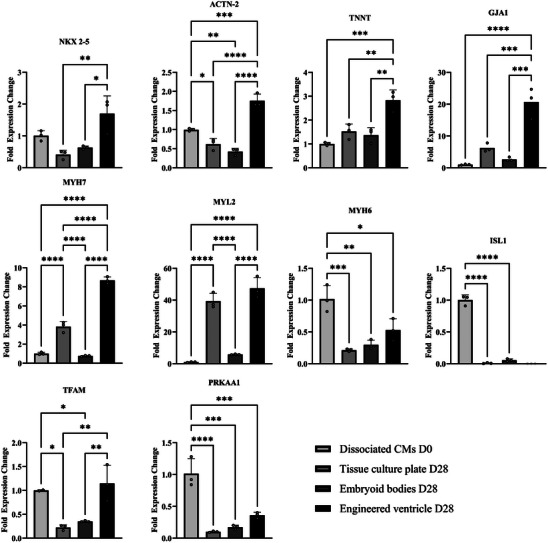
Expression of cardiomyocyte‐related genes, as well as genes associated with gap junctions and mitochondrial activity, in cardiomyocytes from engineered ventricles, tissue culture plates, and CM sphere cultures were compared to those in dissociated cells at day 0. ISL1 expression was not detected in the engineered ventricle group. Statistical analyses were performed using one‐way ANOVA test with *n* = 3 technical replicates for each analysis. Significance levels of ^*^ *p* < 0.05, ^**^ *p* < 0.01, ^***^ *p* < 0.001, and ^****^ *p* < 0.0001 were defined.

The gene expression patterns shown in **Figure** **4** reveal significant upregulation of the cardiomyocyte transcription factor NKX2.5 in the engineered ventricle compared to classical tissue culture and 3D sphere culture. Expression of sarcomere protein‐coding genes such as alpha‐actinin 2 and troponin was also significantly increased compared to the control group at day 0. Notably, the ventricle‐associated genes MYH7 and MYL2 were significantly upregulated by factors of 8.6 and 47.4 respectively in the engineered ventricle group. Conversely, the atrial marker MYH6 and the right heart marker ISL1 were downregulated in all groups at day 28 compared to day 0. ISL1 expression was undetectable in cardiomyocytes from the engineered ventricle. GJA1, associated with Connexin 43 and important for contraction synchronization, was upregulated by a factor of 20.6 compared to the control group. No significant changes in GJA1 expression were observed in the TCP and 3D sphere cultivation groups compared to the control. The mitochondrial biogenesis gene TFAM showed no significant difference in expression in cardiomyocytes from the engineered ventricle compared to the control. However, TFAM expression was significantly downregulated in the TCP and cardiac sphere cultivation groups. The AMP‐activated protein kinase‐associated gene PRKAA1 was significantly downregulated in all groups at day 28 compared to day 0, with no significant differences observed between the engineered ventricle, TCP, and 3D sphere culture groups.

### 3.4 Engineered Ventricle Model with Co‐Culture of Cardiomyocytes with Cardiac Fibroblasts and Cardiac Endothelial Cells

**Figure 5:**
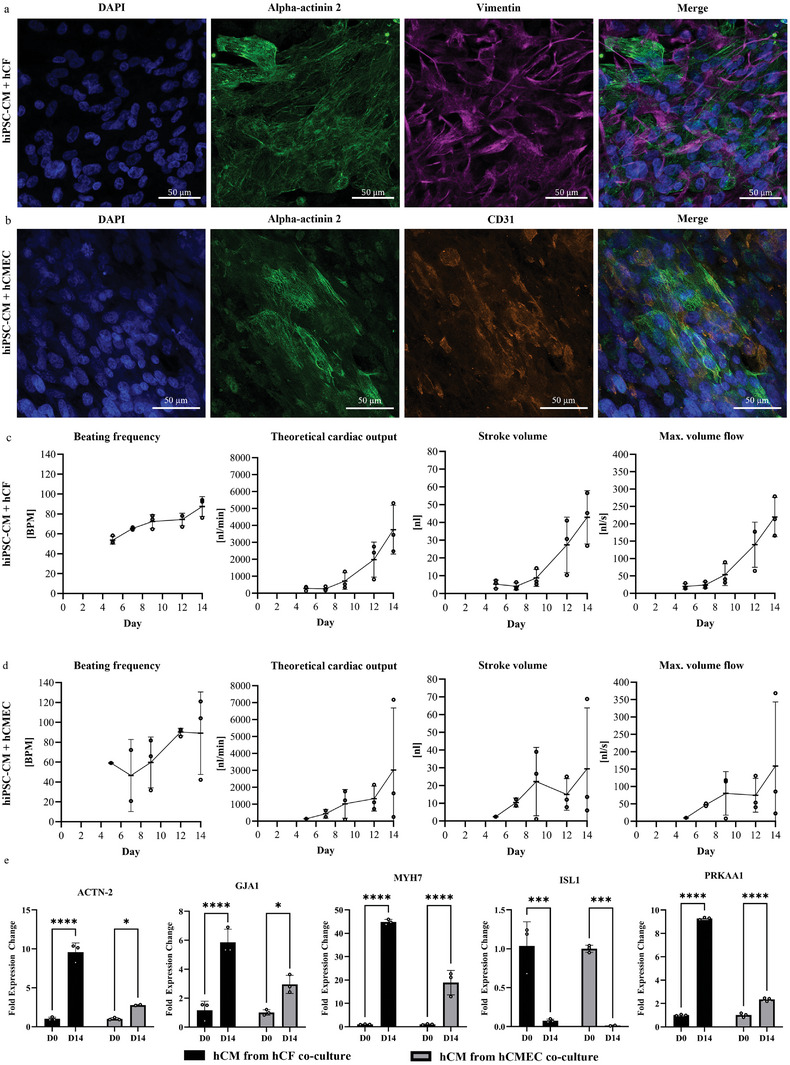
a) Immunofluorescence of co‐culture of hiPSC‐CMs with hCF on engineered ventricles. b) Immunofluorescence of co‐culture of hiPSC‐CMs with hCMEC on engineered ventricles. c) Beating analysis of engineered ventricles seeded with hiPSC‐CMs with hCF. d) Beating analysis of engineered ventricles seeded with hiPSC‐CMs with hCMEC. e) Expression levels of cardiomyocyte‐related genes, as well as genes associated with gap junctions and mitochondrial activity, in cardiomyocytes isolated from engineered ventricles co‐cultured with hCFs and hCMECs using MACS. Statistical analyses were performed using one‐way ANOVA for c) and d) as well as two‐way ANOVA for e). *n* = 3 engineered ventricles at c) and d). *n* = 3 technical replicates for e). Significance levels of ^*^ *p* < 0.05, ^**^ *p* < 0.01, ^***^ *p* < 0.001, and ^****^ *p* < 0.0001 were defined.

Immunofluorescence staining confirmed the successful co‐culture of cardiomyocytes with either cardiac fibroblasts (identified by vimentin staining) or cardiac microvascular endothelial cells (identified by CD31 staining) on the engineered ventricle (**Figure** **5**a,b). A homogeneous distribution of the different cell types across the entire PDMS membrane was observed, as further analyzed regions of interest confirm (Figure S6, Supporting Information).The beating frequency in the co‐culture with hCF and hCMEC was comparable to the monoculture, with 53.6‐87.4 and 46.5–90.2 bpm, respectively (Figure 5c,d). However, theoretical cardiac output, stroke volume, and maximum volume flow were increased in the co‐cultures compared to the hCM monoculture. It should be noted that the co‐culture was maintained in a different medium and could only be cultivated until day 14 due to overgrowth. Over the cultivation period, an increase in all measured parameters from day 5 to 14 was observed. Namely theoretical cardiac output up to 263–3747 nL min^−1^ was measured in the co‐culture with hCF and 148–3020 nL min^−1^ in the co‐culture with hCMEC. Expression levels of cardiomyocyte‐related genes, as well as genes associated with gap junctions and mitochondrial activity, are presented in Figure 5e. The sarcomere protein‐coding gene alpha‐actinin 2, the ventricle‐associated gene MYH7, and GJA1, which encodes Connexin 43, were significantly upregulated in cardiomyocytes isolated from both co‐culture conditions. Notably, the fold‐change in gene expression was approximately 2–3.5 times higher in the hCF co‐culture group compared to the hCMEC co‐culture group. Conversely, the right‐heart marker ISL1 was significantly downregulated in both co‐culture groups. The AMP‐activated protein kinase‐associated gene PRKAA1 showed an upregulation of approximately 9.3‐fold in the hCF co‐culture group and 2.35‐fold in the hCMEC co‐culture group.

### 3.5 Response of the Engineered Ventricle to Cardioactive Drugs

**Figure 6:**
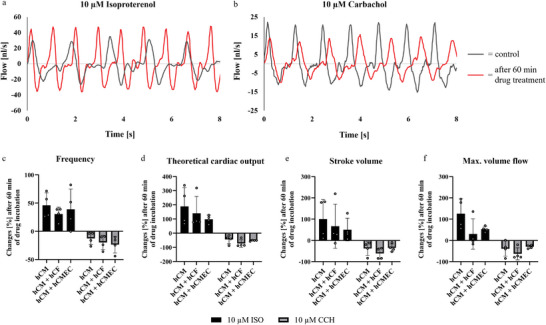
Exemplary response of the engineered ventricles to isoproterenol (ISO) a) and carbachol (CCH) b), and quantification of the beating frequency c), theoretical cardiac output d), stroke volume e), and max. volume flow f) in comparison to pure hCM culture and co‐cultures with hCFs and hCMECs. No significant differences were found by statistical analysis within a drug group. c–f): statistical analyses were performed using two‐way ANOVA with *n* = 4 engineered ventricles, and no significant differences were found between different culture groups of each treatment. Significance levels of ^*^ *p* < 0.05, ^**^ *p* < 0.01, ^***^ *p* < 0.001, and ^****^ *p* < 0.0001 were defined.

Exemplary flow measurements of control and after 60 min of drug incubation of a pure cardiomyocyte seeded engineered ventricle are shown in **Figure** **6**a,b. Notably, both the amplitude of the flow and the beating frequency increased following a 60‐min treatment with isoproterenol. Conversely, treatment with carbachol resulted in a decrease in both flow amplitude and frequency. Quantification of these behaviors is presented in Figure 6c–f, comparing pure cardiomyocyte cultures with co‐cultures containing cardiac fibroblasts as well as cardiac microvascular endothelial cells. The mean frequency increased by 30.6–45.9% following ISO treatment and decreased by 12.6–23.7% after CCH treatment. The effect on theoretical cardiac output was even more pronounced, showing an increase of 98.2–189.1% with ISO and a decrease of 44.0–72.7% with CCH. Similar effects were observed in the measurements of stroke volume and maximum volume flow, although larger standard deviations were noted in some cases (e.g., co‐culture of hCM and hCF). Statistical analysis revealed no significant variations within drug groups across different cell type groups.

## 4 Discussion

The use of in vitro models as an alternative to animal models for the investigation of physiological and pathological relationships as well as the development of therapeutic approaches is becoming increasingly important in research.^[^ ^2^, ^3^, ^4^, ^5^ ^]^ However, models of cardiovascular disease lag behind other organ models,^[^ ^9^ ^]^ often failing to mimic pulsatile flow and thus lacking a fundamental characteristic of human cardiac function.^[^ ^13^, ^14^ ^]^ While there are a few models that can effectively mimic the pumping function of the heart, they are typically associated with significant cost and labor intensity.^[^ ^19^, ^20^ ^]^ Here we explain how our novel in vitro left ventricular model can advance this field of research.

To be able to immobilize cardiomyocytes and other cell types stable on the hemispherical PDMS membrane over a long term, it is essential to achieve hydrolysis‐stable crosslinking between the laminin E8 511 fragments and the PDMS substrate. To achieve this goal, the silane 3MOBS was tested, as well as polydopamine for crosslinking in combination with different pretreatment methods such as UV‐light or acid deposition. The contact angles following the 3MOBS treatments in combination with protein deposition were the highest compared to the polydopamine treatments (Figure 2a), indicating hydrophobic behavior (contact angle > 90°). Brightfield images and immunofluorescence images (Figure 2c; Figure S1, Supporting Information) revealed that cardiomyocytes can adhere to and spread on the 3MOBS‐treated PDMS when combined with laminin E8 511 deposition. Notably, vapor deposition resulted in higher cell density than solvent deposition of 3MOBS. This finding is consistent with studies by Hardelauf et al., which demonstrated that gas‐phase silanization achieved better results than solvent submersion.^[^ ^43^ ^]^ However, since synchronous beating was only observed at low amplitude on day 7 (Figure 2d), we do not recommend using 3MOBS crosslinking for single‐cell cardiomyocyte seeding on PDMS. Goßmann et al. previously used 3MOBS with iPSC‐derived cardiomyocytes cultured for 14 days and concluded that their system was fast, facile, and precise for pharmacological and toxicological studies.^[^ ^44^ ^]^ Nevertheless, it must be considered that different differentiation protocols for cardiomyocytes and variations in dissociation before seeding can lead to differing results. Compared to 3MOBS, it was found that polydopamine significantly outperformed in terms of hydrophilicity, number of attached cells, and contraction behavior. The contact angles after dopamine treatment alone were consistent with measurements reported by Chuah et al. (Figure S3, Supporting Information).^[^ ^31^ ^]^ However, we could not confirm their reported contact angle measurements following complete functionalization with collagen I (approximately 92°).^[^ ^31^ ^]^ Instead, our data showed a mean contact angle of 45.4°. Brightfield images revealed that combining polydopamine treatment with Laminin E8 511 resulted in faster adherence of cardiomyocytes compared to 3MOBS (Figure S1, Supporting Information). Additionally, cell distributions appeared more homogeneous. These observations were further confirmed by immunofluorescence staining. A confluent high‐density layer of cardiomyocytes with a widespread actin cytoskeleton and abundant vinculin, indicating focal adhesion, was visible in all groups using polydopamine for crosslinking (Figure 2c). Notably, quantitative analysis revealed cell densities approximately 5–10 times higher than those observed after 3MOBS functionalization (Figure 2b). Analysis of cell beating demonstrated fully functional cardiomyocytes up to 14 days post‐seeding in PDA‐treated groups (except for the acid + PDA + Laminin E8 group, which could not be analyzed by video due to its non‐transparency). The UV‐light + PDA + Laminin E8 treatment group exhibited a mean beating frequency of 65 bpm, which falls within the physiological range^[^ ^45^ ^]^ and is comparable to results achieved by Etezadi et al., who differentiated iPSCs into cardiomyocytes on a PDA and Laminin E8 coated PDMS surface.^[^ ^46^ ^]^ Consequently, we recommend combined surface treatment of PDMS with dopamine and Laminin E8 fragments as an ideal functionalization method for reseeding single‐cell suspensions of cardiomyocytes. This method was also used to further develop our engineered ventricle model.

The next step was to demonstrate that cardiomyocytes seeded on a hemispherical membrane can create a pulsatile flow by contracting the membrane's surface and thereby displacing the culture medium inside. Between days 5 and 7, a flow was measurable, indicating that the cardiomyocytes in the engineered ventricles had attached to the membrane and begun to beat synchronously. Additionally, we showed that other cell types, such as cardiac fibroblasts and cardiac microvascular endothelial cells, attach to the biofunctionalized PDMS membrane in co‐culture with the cardiomyocytes, as evidenced by immunofluorescence staining (Figure 5a,b). This underscores the ability to create a more physiological model by mimicking cell–cell interactions among different cell types in the myocardium. Notably, to achieve a physiologically consistent model, cells were used that were only from donors of the same gender due to differences in cellular phenotype, behavior, and composition between male and female hearts.^[^ ^41^ ^]^ We have identified three possible reasons for the increase through day 14 in the flow generated by the engineered ventricle, characterized by theoretical cardiac output, stroke volume, and maximum volume flow. The first reason is the ongoing cardiac differentiation. Cardiomyocytes seeded on day 0 are relatively immature and continue to express more sarcomere proteins over time. Second, following the harsh dissociation conditions, the cells need several days to recover and rebuild their surface proteins for attachment and gap junctions for synchronized beating. The third possibility could be a combination of these aforementioned reasons. After day 14, it was only possible to cultivate the monoculture of cardiomyocytes (CMs), as the co‐cultures overgrew and detached from the membrane. In the future, custom‐designed culture media could be used to circumvent the problem of stromal cell overgrowth. However, the theoretical cardiac output generated by the engineered ventricles with the monoculture of CMs tended to decrease until day 28. This phenomenon was unexpected, as Hamad et al. demonstrated stable beating characteristics of iPSC‐derived CMs from day 20 to 40^[^ ^17^ ^]^ (equivalent to our counting from day 10 to 30). Nevertheless, it should be generally noted that the observed trends are not statistically significant in the group of pure cardiomyocytes and should therefore not be overestimated. Compared to the bioprinted ventricle from Lee et al., which showed a beating rate of 30 bpm,^[^ ^19^ ^]^ our ventricle model exhibits more physiological frequencies ranging between 46.5 and 97.47 bpm across all tested groups. Additionally, Lee's ventricle model was not integrated into a bioreactor that supported measurements of the generated flow. MacQueen et al. generated a ventricle model by seeding human cardiomyocytes onto an electrospun ventricle scaffold and measured a volume change during a contraction cycle of approximately 0.4 µL.^[^ ^20^ ^]^ This is about ten times higher than the maximum mean stroke volume of 43 nl measured in our engineered ventricle. This difference is likely due to their use of less stiff and thinner support material compared to our 100 µm thick PDMS membrane. We validated this hypothesis by fabricating PDMS membranes with double the thickness (200 µm), which resulted in a stroke volume and theoretical cardiac output reduced by half, while the beating frequency remained unaffected (Figure S5, Supporting Information). The mean beating frequency in their model was about 85 bpm,^[^ ^20^ ^]^ which is comparable to our engineered ventricles. However, they only cultivated pure cardiomyocytes within 14 days after seeding. Another approach to achieving a cardiac pump involved using two‐photon direct laser writing (TPDLW) to fabricate a nanoscale‐resolution metamaterial scaffold, which was then seeded with cardiomyocytes. This method resulted in an ejected volume of about 60 nl,^[^ ^47^ ^]^ comparable to our engineered ventricle. It is noteworthy that all of the models discussed here can only be produced with high cost and labor input (3D bioprinting, electrospinning, two‐photon direct laser writing). In comparison, our ventricle model is produced simply by casting PDMS into a hemispherical shape and seeding it with a single‐cell suspension.

To further characterize the cell cultivation conditions on the hemispherical membrane, we performed flow cytometry and qPCR analysis in comparison to classical tissue culture plates and 3D sphere cultivation. Using flow cytometry, we investigated alpha‐actinin 2 expression in cardiomyocytes on day 0 and day 28 under the three different culture conditions. All groups exhibited over 97.7% positive alpha‐actinin 2 expression (Figure 3d), with no clear differences observed between the different culture conditions. Analyzing cardiac gene expressions provided more precise insights. Cardiomyocytes from the engineered ventricle exhibited significantly higher expression of the cardiac transcription factor NKX‐2.5 compared to cells from tissue culture plates and 3D sphere cultures (Figure 4). Additionally, genes coding for sarcomere proteins alpha‐actinin 2 and cardiac troponin T were significantly upregulated. Notably, the left ventricular isoforms MYH7 and MYL2^[^ ^48^ ^]^ were massively upregulated in the engineered ventricle culture, whereas the atrial isoform MYH6^[^ ^48^ ^]^ was downregulated, and the early stage heart marker ISL1^[^ ^48^ ^]^ was undetectable. Taken together, these data support that the assertion that hemispherical PDMS membrane provides an ideal culture condition for the differentiation and maintenance of human left ventricular cardiomyocytes. We suspect that one reason for this improved differentiation is that the substrate has a stiffness similar to that of human myocardium.^[^ ^28^, ^29^ ^]^ This allows it to be contracted by cardiomyocytes while still offering a certain amount of resistance. Moreover, the connexin 43‐associated GJA1 gene is significantly upregulated compared to the control and standard cultivation methods. As a result, cardiomyocytes on the engineered ventricle will have better cell‐cell connections, which are important for synchronous contraction. On the other hand, the mitochondrial biogenesis gene TFAM showed no significant difference in expression in cardiomyocytes from the engineered ventricle compared to the control. Additionally, the AMP‐activated protein kinase‐associated gene PRKAA1 was significantly downregulated in all groups on day 28 compared to day 0. We had expected an increase during differentiation and maturation of the cardiomyocytes, as increased mitochondrial development should accompany the transition to oxidative phosphorylation. One possible explanation for this observation could be that the culture medium used contains only a low concentration of fatty acids and therefore does not support these processes. Similar processes were described by Chen et al.^[^ ^49^ ^]^

To further investigate time‐dependent trends in gene expression, we conducted qPCR analysis of gene expression in cardiomyocytes from the engineered ventricle on day 14 (Figure S7, Supporting Information). The analysis revealed that genes encoding sarcomere proteins, such as ACTN2, TNNT, and MYL2, were upregulated from day 0 to 14, followed by a decline through day 28. Despite this decline, expression levels remained higher than those observed on day 0. A similar pattern was observed for NKX‐2.5 and TFAM. These findings align with the observations of Zhou et al., who reported a significant increase in cardiac gene expression from day 0 to 20 (corresponding to days −10 to 10 in our timeline) and a subsequent decrease between day day 30 and 60 (days 20 to 50 in our timeline), coinciding with protein maturation.^[^ ^50^ ^]^ This trend was further validated by data from Grancharova et al.^[^ ^51^ ^]^

The qPCR analysis of cardiac‐related genes in CMs isolated via MACS from the two co‐cultures (Figure 5e) revealed significant upregulation from day 0 to 14. Combined with the higher stroke volume and theoretical cardiac output observed in the co‐culture groups compared to the CM monoculture, these findings align with the literature: hCF and hCMEC significantly enhance hCM function through paracrine signaling, secreting growth factors, cytokines, and ECM components. hCF contributes to structural integrity and contractility by producing ECM proteins, such as collagen and fibronectin, which support cellular organization and mechanical stability.^[^ ^52^, ^53^, ^54^ ^]^ Moreover hCMEC supports efficient metabolic and electrical function by increasing oxygenation and electrophysiological properties through the release of vascular endothelial growth factor (VEGF) and nitric oxide.^[^ ^55^, ^56^ ^]^ Both hCF and hCMEC also promote structural maturation. hCF facilitates alignment and organization of hCMs by remodeling the ECM, while hCMEC promotes tissue‐like microenvironments, contributing to the formation of capillary‐like networks. Electrical coupling is further improved because hCF enables synchronized contractions by expressing gap junction proteins such as connexins, and hCMEC regulates ion channel activity, contributing to more efficient conduction. In addition, coculture may improve hCM survival and limited proliferation, increasing the number of functional cardiomyocytes.^[^ ^57^, ^58^, ^59^ ^]^

Another important aspect of the ventricle model is whether it reacts as anticipated to sympathetic and parasympathetic stimuli from cardioactive drugs. Notably, we were able to demonstrate effects not only on the beating frequency but also on other key parameters such as theoretical cardiac output, stroke volume, and maximum volume flow (Figure 6). Starting with the frequency changes, we can confirm data showing an increase of around 88% after 10 µM ISO treatment reported by Hamad et al.^[^ ^17^ ^]^ and around 15 % reported by MacQueen et al.,^[^ ^20^ ^]^ with measurements showing an increase of 30.6 to 45.9%. We can also confirm the frequency decrease after 10 µM CCH treatment of around 40%,^[^ ^17^ ^]^ with our measurements showing a decrease of about 12.6 to 23.7% in the engineered ventricles. In addition, the effects on stroke volume resulting from changes in contraction forces were measured. As anticipated, the stroke volume increased following ISO treatment and decreased following CCH treatment. Since cardiac output is the mathematical product of beat frequency and stroke volume, the effects of ISO and CCH are even more pronounced when raised to the second power. Specifically, theoretical cardiac output increased by approximately 98.2 to 189.1% with ISO treatment and decreased by approximately 44–72.7% with CCH incubation. In summary, these data reveal that the engineered ventricle could serve as an in vitro model of the human left ventricle for simple drug screenings.

Another potential application of the engineered ventricle model is its use in body‐on‐a‐chip systems. When coupled with two check valves, as demonstrated by Tanaka et al.,^[^ ^60^ ^]^ the ventricle can not only serve as an organ model but also power entire body‐on‐a‐chip systems. This setup would allow for direct feedback of drugs or cytokines, resulting in changes in the medium flow also for the other connected organ models. While this study establishes the engineered ventricle as a functional in vitro model for drug testing, we recognize that it is not yet tailored for patient‐specific applications. The current model does not account for the genetic or phenotypic variability of patient‐derived cells. However, its modular design provides a basis for future adaptations. By integrating cardiomyocytes derived from patient‐specific hiPSCs or incorporating disease‐relevant modifications, the model could be extended to study personalized therapeutic responses and pathophysiological mechanisms.

## 5 Conclusion

Our study demonstrates that a hemispherical PDMS membrane biofunctionalized with dopamine and laminin E8 provides an optimal environment for the long‐term culture and differentiation of human left ventricular cardiomyocytes. The engineered ventricle model not only supports synchronous contraction and physiological beating frequencies but also responds effectively to sympathetic and parasympathetic stimuli, making it a robust in vitro model for drug screening. It´s flexibility concerning the seeded cell types it might serve in the future as patient‐specific applications by incorporating disease associated cell types and patient‐specific hiPSCs. In addition, its potential integration into body‐on‐a‐chip systems could offer significant advances in organ modeling and pharmacological testing, by directly coupling the cardiac output in response to cellular response to other organ models. The simplicity and cost‐effectiveness of fabricating this ventricle model further underscores its applicability for various biomedical research applications.

## Conflict of Interest

The authors declare no conflict of interest.

## Supporting information



Supporting Information

Supplemental Video 1

Supplemental Video 2

Supplemental Video 3

## Data Availability

The data that support the findings of this study are available from the corresponding author upon reasonable request.
