# Supplementary material for: Engineered In Vitro Multi‐Cell Type Ventricle Model Generates Long‐Term Pulsatile Flow and Modulates Cardiac Output in Response to Cardioactive Drugs
Source: Adv Healthc Mater. 2025 Feb 13;14(10):2403897. doi: 10.1002/adhm.202403897 (PMC12004430; doi:10.1002/adhm.202403897)
Supplement: Supplementary file 1 — Supporting Information [file ADHM-14-0-s001.docx]

**Engineered in vitro multi-cell type ventricle model generates long-term pulsatile flow and modulates cardiac output in response to cardioactive drugs**

Christoph Kuckelkorn, Ebru Aksoy, Natalija Stojanovic, Laila Oulahyane, Mira Ritter, Kurt Pfannkuche, Horst Fischer*

C. Kuckelkorn

Department of Dental Materials and Biomaterials Research, RWTH Aachen University Hospital, Pauwelsstrasse 30, 52074 Aachen, Germany

E. Aksoy

Center for Physiology and Pathophysiology, Institute for Neurophysiology, University and University Hospital of Cologne, Robert Koch Str. 39, 50931 Cologne, Germany

N. Stojanovic

Department of Dental Materials and Biomaterials Research, RWTH Aachen University Hospital, Pauwelsstrasse 30, 52074 Aachen, Germany

L. Oulahyane

Department of Dental Materials and Biomaterials Research, RWTH Aachen University Hospital, Pauwelsstrasse 30, 52074 Aachen, Germany

M. Ritter

Department of Dental Materials and Biomaterials Research, RWTH Aachen University Hospital, Pauwelsstrasse 30, 52074 Aachen, Germany

Prof. Dr. rer. med. K. Pfannkuche

1.) Center for Physiology and Pathophysiology, University and University Hospital of Cologne, Robert Koch Str. 39, 50931 Cologne, Germany

2.) Center for Molecular Medicine Cologne (CMMC), Cologne, Germany

3.) Marga-and-Walter-Boll-Laboratory for Cardiac Tissue Engineering, Cologne, Germany

Univ.-Prof. Dr.-Ing. H. Fischer

Department of Dental Materials and Biomaterials Research, RWTH Aachen University Hospital, Pauwelsstrasse 30, 52074 Aachen, Germany

Email: hfischer@ukaachen.de

*Corresponding author

**Supporting Information**

**Supplementary Table 1**. List of used antibodies for immunofluorescence staining as well as flow cytometry.

| **Antibody** | **Manufacturer** | **Dilution** |
| --- | --- | --- |
| Alexa Fluor 488 Phalloidin, A12379 | Thermo Fisher Scientific, Waltham, USA | 1:250 |
| Vinculin, 14-9777-82 | Thermo Fisher Scientific, Waltham, USA | 1:5000 |
| Alpha Actinin 2, 701914 | Thermo Fisher Scientific, Waltham, USA | 1:500 |
| Troponin T-C, sc-20025 | Santa Cruz Biotechnology, Dallas, USA | 1:250 |
| Vimentin V 9, sc-6260 | Santa Cruz Biotechnology, Dallas, USA | 1:250 |
| PECAM-1/CD31, sc-376764 | Santa Cruz Biotechnology, Dallas, USA | 1:250 |
| Alexa Fluor 555, A-31570 | Thermo Fisher Scientific, Waltham, USA | 1:1000 |
| Alexa Fl. 488, A2515207 | Thermo Fisher Scientific, Waltham, USA | 1:1000 |
| Brilliant Violet 421, 406616 | BioLegend, San Diego, USA | 1:1000 |

**Supplementary Table 2.** qPCR Reaction mix components, per one reaction with total volume 10 µL.

| **Component** | **Volume per reaction [µL]** |
| --- | --- |
| SYBR Green | 5 |
| Primer forward 10 µM | 0.5 |
| Primer reverse 10 µM | 0.5 |
| RNase-free water | 3 |
| cDNA or Water (non-template control) | 1 |

**Supplementary Table 3.** Primer sequences

| GAPDH forward | CAAGGTCATCCATGACAACTT TG |
| --- | --- |
| GAPDH reverse | GTCCACCACCCTGTTGCTGTA G |
| NKX 2,5 forward | ACCTCAACAGCTCCCTGACTCT |
| NKX 2,5 reverse | ATAATCGCCGCCACAAACTCTCC |
| ACTN2 forward | TCGTGAACACCCCTAAACCC |
| ACTN2 reverse | CGCAAAAGCGTGGTAGAAGC |
| TNNT 2 forward | ACAGAGCGGAAAAGTGGGAAG |
| TNNT 2 reverse | TCGTTGATCCTGTTTCGGAGA |
| MYL 2 forward | TTGGGCGAGTGAACGTGAAAA |
| MYL 2 reverse | CCGAACGTAATCAGCCTTCAG |
| MYH 7 forward | CTTTGCTGTTATTGCAGCCATT |
| MYH 7 reverse | AGATGCCAACTTTCCTGTTGC |
| TFAM forward | CGCTCCCCCTTCAGTTTTGT |
| TFAM reverse | CCAACGCTGGGCAATTCTTC |
| PRKAA1 forward | CGGAGCCTTGATGTGGTAGG |
| PRKAA1 reverse | AGATGGTGTACTGATGACCTGG |
| ISL1 forward | CTTTCAGCATTGGCAACCCC |
| ISL1 reverse | GATTGCCGCAACCAACACAT |
| MYH6 forward | CCGTGAAGGGATAACCAGGG |
| MYH6 reverse | TCTTCCTTGTCATCGGGCAC |
| GJA1 forward | AGCCACTAGCCATTGTGGAC |
| GJA1 reverse | CCACCTCCACCGGATCAAAA |

**
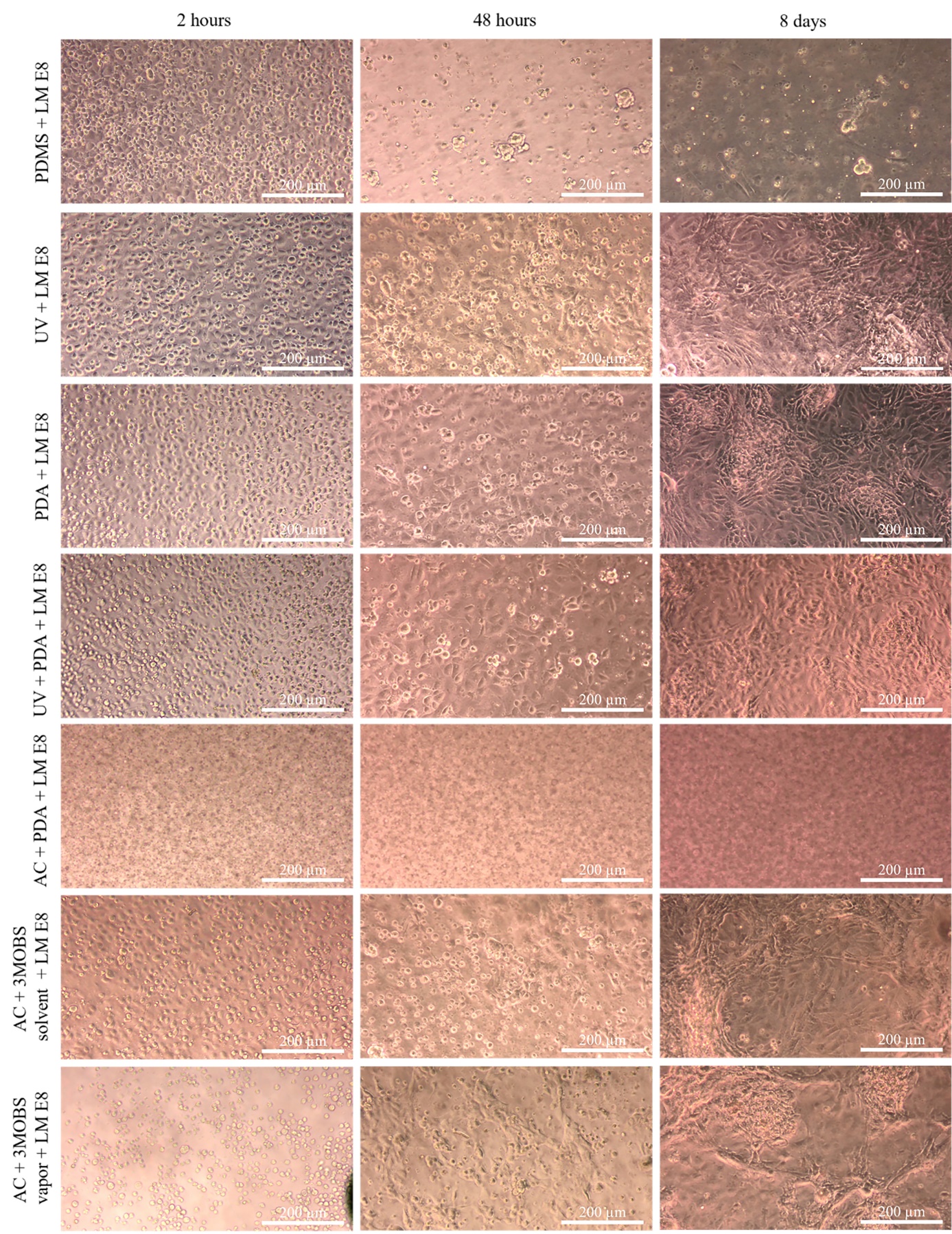
**

**Supplementary Figure 1.** Brightfield microscopy of hiPSC derived cardiomyocytes on different fuctionalization methods for PDMS at 2hours, 48 hours and 8 days after seeding. Scale bar = 200µm.


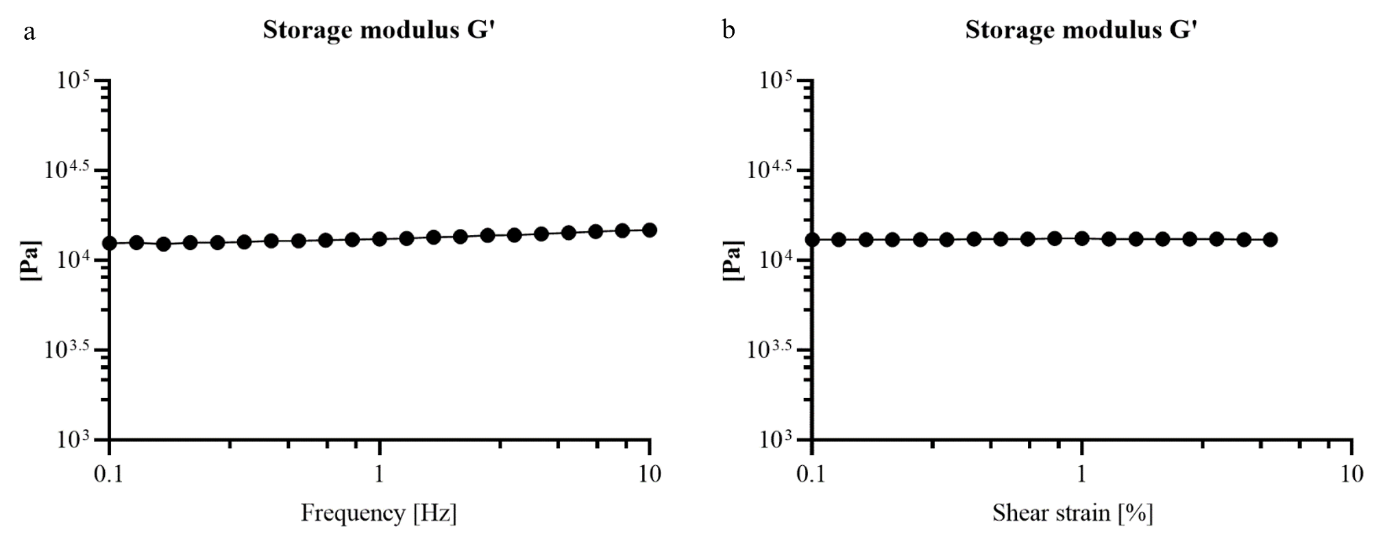


**Supplementary Figure 2.** Storage modulus G’ of PDMS mixed in a ratio of 25:1 (base : curing agent) and cured at 60°C depending on a) fruquency at constant strain (0,5 %) and b) shear strain at constant frequency (1 Hz). Measurement performed at rotational rheometers (Kinexus, Malvern Instruments).

**Supplementary Table 4.** Not shown significances of cardiomyocyte count by DAPI signal after different functionalization methods of PDMS from figure 2b.

| Tukey's multiple comparisons test | Mean Diff, | 95,00% CI of diff, | Below threshold? | Summary | Adjusted P Value |
| --- | --- | --- | --- | --- | --- |
| PDMS + LM E8 vs. UV + LM E8 | -25322 | -164759 to 114114 | No | ns | 0,9949 |
| PDMS + LM E8 vs. PDA + LM E8 | -440284 | -579721 to -300847 | Yes | **** | <0,0001 |
| PDMS + LM E8 vs. UV + PDA + LM E8 | -470508 | -609944 to -331071 | Yes | **** | <0,0001 |
| PDMS + LM E8 vs. AC + PDA + LM E8 | -432115 | -571552 to -292679 | Yes | **** | <0,0001 |
| PDMS + LM E8 vs. AC+ 3MOBS solvent + LM E8 | -25322 | -164759 to 114114 | No | ns | 0,9949 |
| PDMS + LM E8 vs. AC + 3MOBS vapor + LM E8 | -103740 | -243177 to 35697 | No | ns | 0,2168 |
| UV + LM E8 vs. PDA + LM E8 | -414962 | -554398 to -275525 | Yes | **** | <0,0001 |
| UV + LM E8 vs. UV + PDA + LM E8 | -445185 | -584622 to -305748 | Yes | **** | <0,0001 |
| UV + LM E8 vs. AC + PDA + LM E8 | -406793 | -546230 to -267356 | Yes | **** | <0,0001 |
| UV + LM E8 vs. AC+ 3MOBS solvent + LM E8 | 0 | -139437 to 139437 | No | ns | >0,9999 |
| UV + LM E8 vs. AC + 3MOBS vapor + LM E8 | -78418 | -217855 to 61019 | No | ns | 0,4993 |
| PDA + LM E8 vs. UV + PDA + LM E8 | -30224 | -169660 to 109213 | No | ns | 0,9872 |
| PDA + LM E8 vs. AC + PDA + LM E8 | 8169 | -131268 to 147605 | No | ns | >0,9999 |
| PDA + LM E8 vs. AC+ 3MOBS solvent + LM E8 | 414962 | 275525 to 554398 | Yes | **** | <0,0001 |
| PDA + LM E8 vs. AC + 3MOBS vapor + LM E8 | 336544 | 197107 to 475981 | Yes | **** | <0,0001 |
| UV + PDA + LM E8 vs. AC + PDA + LM E8 | 38392 | -101045 to 177829 | No | ns | 0,959 |
| UV + PDA + LM E8 vs. AC+ 3MOBS solvent + LM E8 | 445185 | 305748 to 584622 | Yes | **** | <0,0001 |
| UV + PDA + LM E8 vs. AC + 3MOBS vapor + LM E8 | 366767 | 227330 to 506204 | Yes | **** | <0,0001 |
| AC + PDA + LM E8 vs. AC+ 3MOBS solvent + LM E8 | 406793 | 267356 to 546230 | Yes | **** | <0,0001 |
| AC + PDA + LM E8 vs. AC + 3MOBS vapor + LM E8 | 328375 | 188938 to 467812 | Yes | **** | <0,0001 |
| AC+ 3MOBS solvent + LM E8 vs. AC + 3MOBS vapor + LM E8 | -78418 | -217855 to 61019 | No | ns | 0,4993 |

**Supplementary Table 5.** Not shown significances of contact angle after different functionalization methods of PDMS from figure 2c.

| Šídák's multiple comparisons test | Mean Diff, | 95,00% CI of diff, | Below threshold? | Summary | Adjusted P Value |
| --- | --- | --- | --- | --- | --- |
|  |  |  |  |  |  |
| Day 1 |  |  |  |  |  |
|  |  |  |  |  |  |
| PDMS + Protein (a) vs. UV + Protein (b) | 18,9 | 0,7885 to 37,01 | Yes | * | 0,0329 |
| PDMS + Protein (a) vs. PDA + Protein (c) | 1,989 | -16,12 to 20,10 | No | ns | >0,9999 |
| PDMS + Protein (a) vs. UV + PDA + Protein (d) | 5,522 | -12,59 to 23,63 | No | ns | 0,9999 |
| PDMS + Protein (a) vs. AC + PDA + Protein (e) | 3,267 | -14,84 to 21,38 | No | ns | >0,9999 |
| PDMS + Protein (a) vs. Group F | -58,94 | -77,06 to -40,83 | Yes | **** | <0,0001 |
| PDMS + Protein (a) vs. AC + 3MOBS vapor + Protein (g) | -54,59 | -72,70 to -36,48 | Yes | **** | <0,0001 |
| UV + Protein (b) vs. PDA + Protein (c) | -16,91 | -35,02 to 1,200 | No | ns | 0,0914 |
| UV + Protein (b) vs. UV + PDA + Protein (d) | -13,38 | -31,49 to 4,734 | No | ns | 0,3978 |
| UV + Protein (b) vs. AC + PDA + Protein (e) | -15,63 | -33,74 to 2,478 | No | ns | 0,165 |
| UV + Protein (b) vs. Group F | -77,84 | -95,96 to -59,73 | Yes | **** | <0,0001 |
| UV + Protein (b) vs. AC + 3MOBS vapor + Protein (g) | -73,49 | -91,60 to -55,38 | Yes | **** | <0,0001 |
| PDA + Protein (c) vs. UV + PDA + Protein (d) | 3,533 | -14,58 to 21,64 | No | ns | >0,9999 |
| PDA + Protein (c) vs. AC + PDA + Protein (e) | 1,278 | -16,83 to 19,39 | No | ns | >0,9999 |
| PDA + Protein (c) vs. Group F | -60,93 | -79,04 to -42,82 | Yes | **** | <0,0001 |
| PDA + Protein (c) vs. AC + 3MOBS vapor + Protein (g) | -56,58 | -74,69 to -38,47 | Yes | **** | <0,0001 |
| UV + PDA + Protein (d) vs. AC + PDA + Protein (e) | -2,256 | -20,37 to 15,86 | No | ns | >0,9999 |
| UV + PDA + Protein (d) vs. Group F | -64,47 | -82,58 to -46,36 | Yes | **** | <0,0001 |
| UV + PDA + Protein (d) vs. AC + 3MOBS vapor + Protein (g) | -60,11 | -78,22 to -42,00 | Yes | **** | <0,0001 |
| AC + PDA + Protein (e) vs. Group F | -62,21 | -80,32 to -44,10 | Yes | **** | <0,0001 |
| AC + PDA + Protein (e) vs. AC + 3MOBS vapor + Protein (g) | -57,86 | -75,97 to -39,74 | Yes | **** | <0,0001 |
| Group F vs. AC + 3MOBS vapor + Protein (g) | 4,356 | -13,76 to 22,47 | No | ns | >0,9999 |
|  |  |  |  |  |  |
| Day 21 |  |  |  |  |  |
| PDMS + Protein (a) vs. UV + Protein (b) | 23,1 | 4,988 to 41,21 | Yes | ** | 0,0028 |
| PDMS + Protein (a) vs. PDA + Protein (c) | 24,19 | 6,077 to 42,30 | Yes | ** | 0,0014 |
| PDMS + Protein (a) vs. UV + PDA + Protein (d) | 29,42 | 11,31 to 47,53 | Yes | **** | <0,0001 |
| PDMS + Protein (a) vs. AC + PDA + Protein (e) | 25,39 | 7,277 to 43,50 | Yes | *** | 0,0006 |
| PDMS + Protein (a) vs. Group F | -22,34 | -40,46 to -4,233 | Yes | ** | 0,0045 |
| PDMS + Protein (a) vs. AC + 3MOBS vapor + Protein (g) | -17,76 | -35,87 to 0,3560 | No | ns | 0,06 |
| UV + Protein (b) vs. PDA + Protein (c) | 1,089 | -17,02 to 19,20 | No | ns | >0,9999 |
| UV + Protein (b) vs. UV + PDA + Protein (d) | 6,322 | -11,79 to 24,43 | No | ns | 0,999 |
| UV + Protein (b) vs. AC + PDA + Protein (e) | 2,289 | -15,82 to 20,40 | No | ns | >0,9999 |
| UV + Protein (b) vs. Group F | -45,44 | -63,56 to -27,33 | Yes | **** | <0,0001 |
| UV + Protein (b) vs. AC + 3MOBS vapor + Protein (g) | -40,86 | -58,97 to -22,74 | Yes | **** | <0,0001 |
| PDA + Protein (c) vs. UV + PDA + Protein (d) | 5,233 | -12,88 to 23,34 | No | ns | >0,9999 |
| PDA + Protein (c) vs. AC + PDA + Protein (e) | 1,2 | -16,91 to 19,31 | No | ns | >0,9999 |
| PDA + Protein (c) vs. Group F | -46,53 | -64,64 to -28,42 | Yes | **** | <0,0001 |
| PDA + Protein (c) vs. AC + 3MOBS vapor + Protein (g) | -41,94 | -60,06 to -23,83 | Yes | **** | <0,0001 |
| UV + PDA + Protein (d) vs. AC + PDA + Protein (e) | -4,033 | -22,14 to 14,08 | No | ns | >0,9999 |
| UV + PDA + Protein (d) vs. Group F | -51,77 | -69,88 to -33,66 | Yes | **** | <0,0001 |
| UV + PDA + Protein (d) vs. AC + 3MOBS vapor + Protein (g) | -47,18 | -65,29 to -29,07 | Yes | **** | <0,0001 |
| AC + PDA + Protein (e) vs. Group F | -47,73 | -65,84 to -29,62 | Yes | **** | <0,0001 |
| AC + PDA + Protein (e) vs. AC + 3MOBS vapor + Protein (g) | -43,14 | -61,26 to -25,03 | Yes | **** | <0,0001 |
| Group F vs. AC + 3MOBS vapor + Protein (g) | 4,589 | -13,52 to 22,70 | No | ns | >0,9999 |


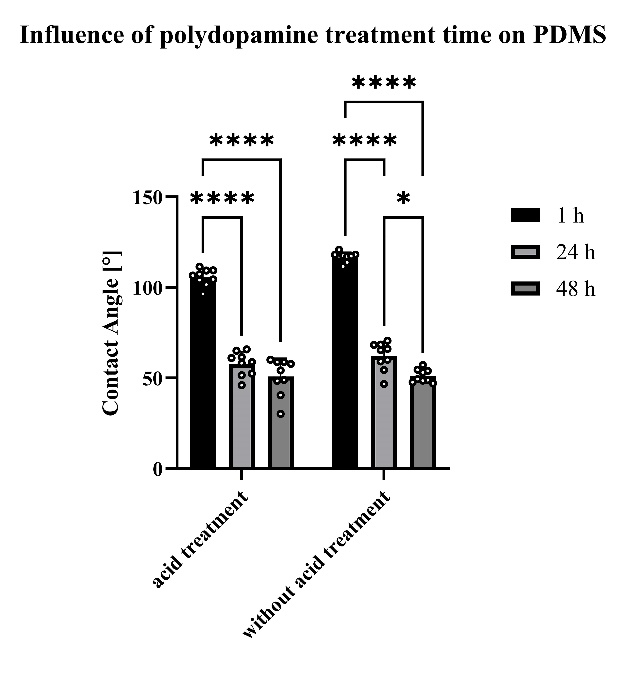


**Supplementary Figure 3.** Influence of polydopamine treatment time in combination without and with a prior acid treatment. Acid used:1 part hydrogen peroxide 30% and 1 part hydrochloric acid 37%. Statistical analyses were performed using two-way ANOVA and n=9 technical replicates. Significance levels of p <0.05 (*), p <0.01 (**), p <0.001 (***) and p <0.0001 (****) were defined.

**
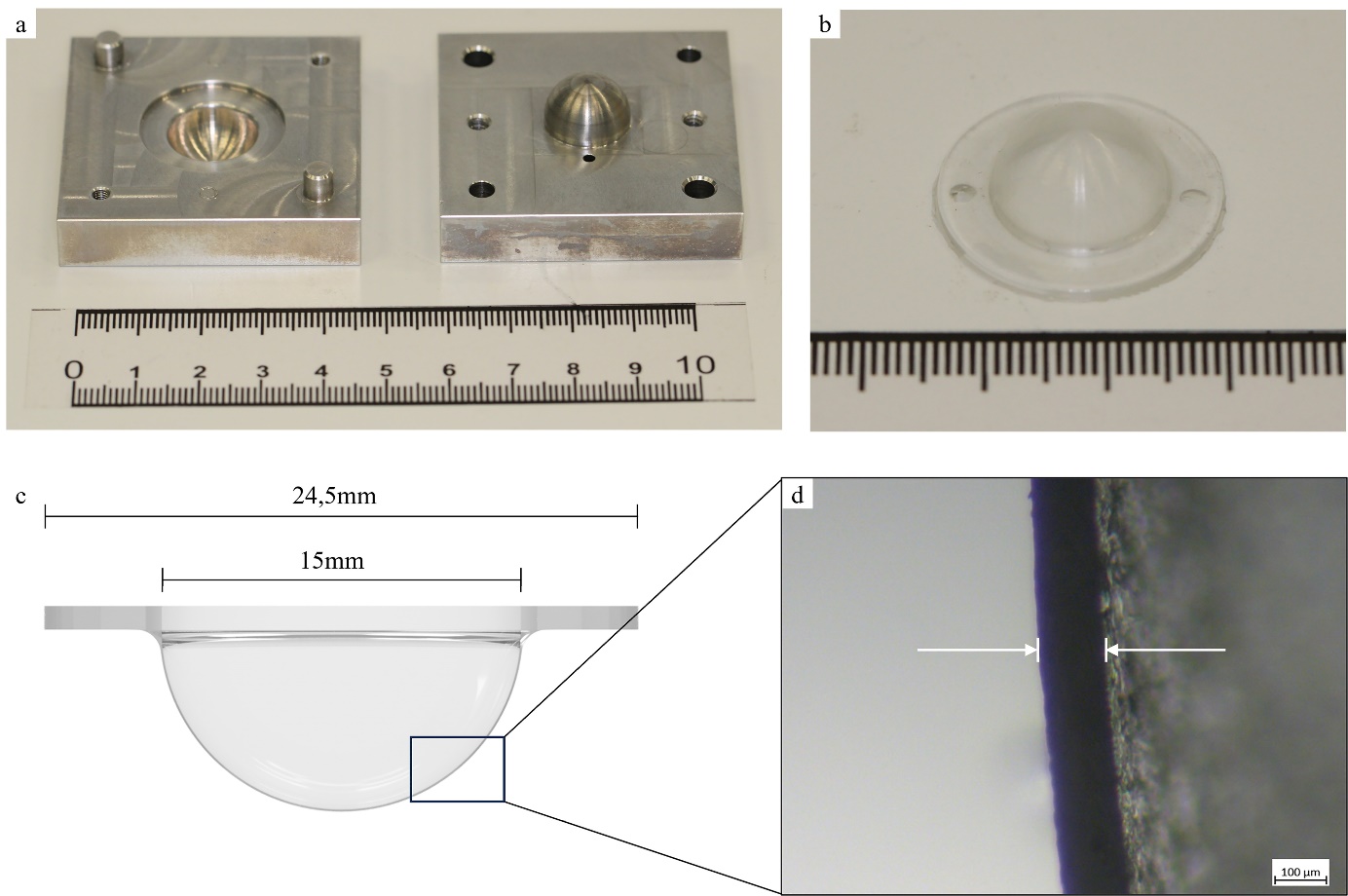
**

**Supplementary Figure 4.** a) Milled aluminum molds for the hemispheric PDMS membrane. b) resulting PDMS membrane. c) PDMS membrane render with geometry and cross-section d) for validating the membrane thickness. Thickness was measured (white arrows) to mean=115,7 μm with standard deviation=18,7 μm.


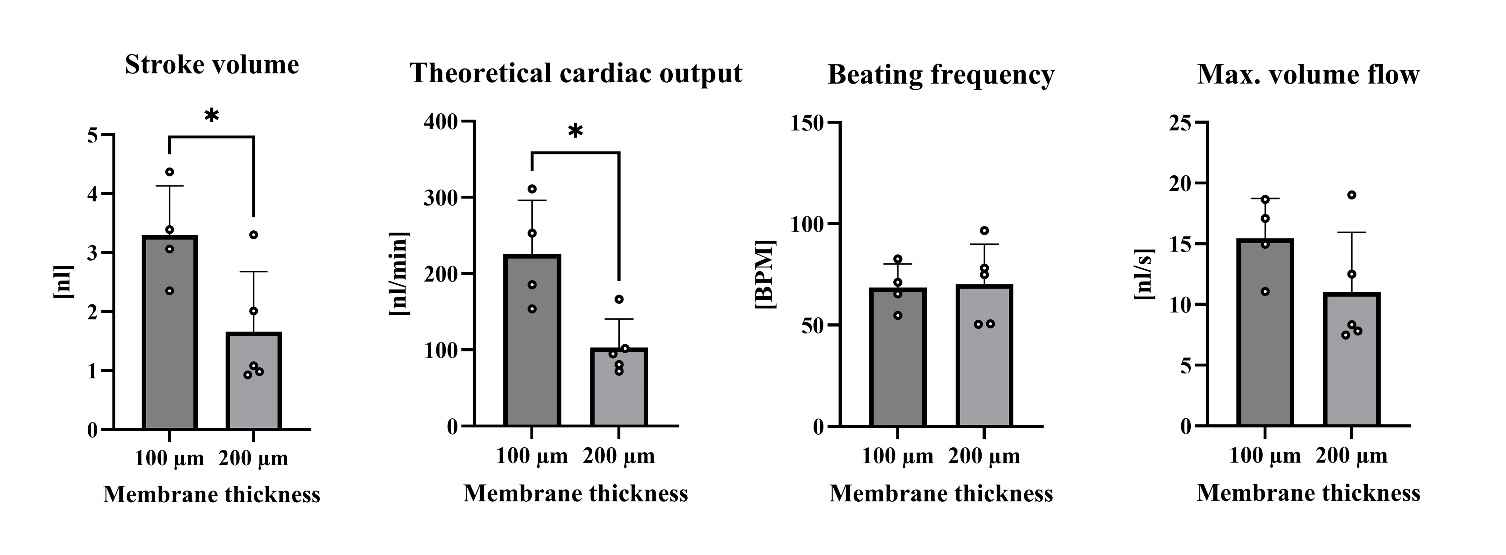


**Supplementary Figure 5:** Comparison of the beating characteristics (stroke volume, theoretical cardiac output, beating frequency, and maximum volumetric flow rate) of cardiomyocyte cultures seeded on PDMS membranes of varying thicknesses, evaluated on day 7 post-seeding. Statistical analyses were performed using T-Test and n=5 engineered ventricles. Significance levels of p <0.05 (*), p <0.01 (**), p <0.001 (***) and p <0.0001 (****) were defined.


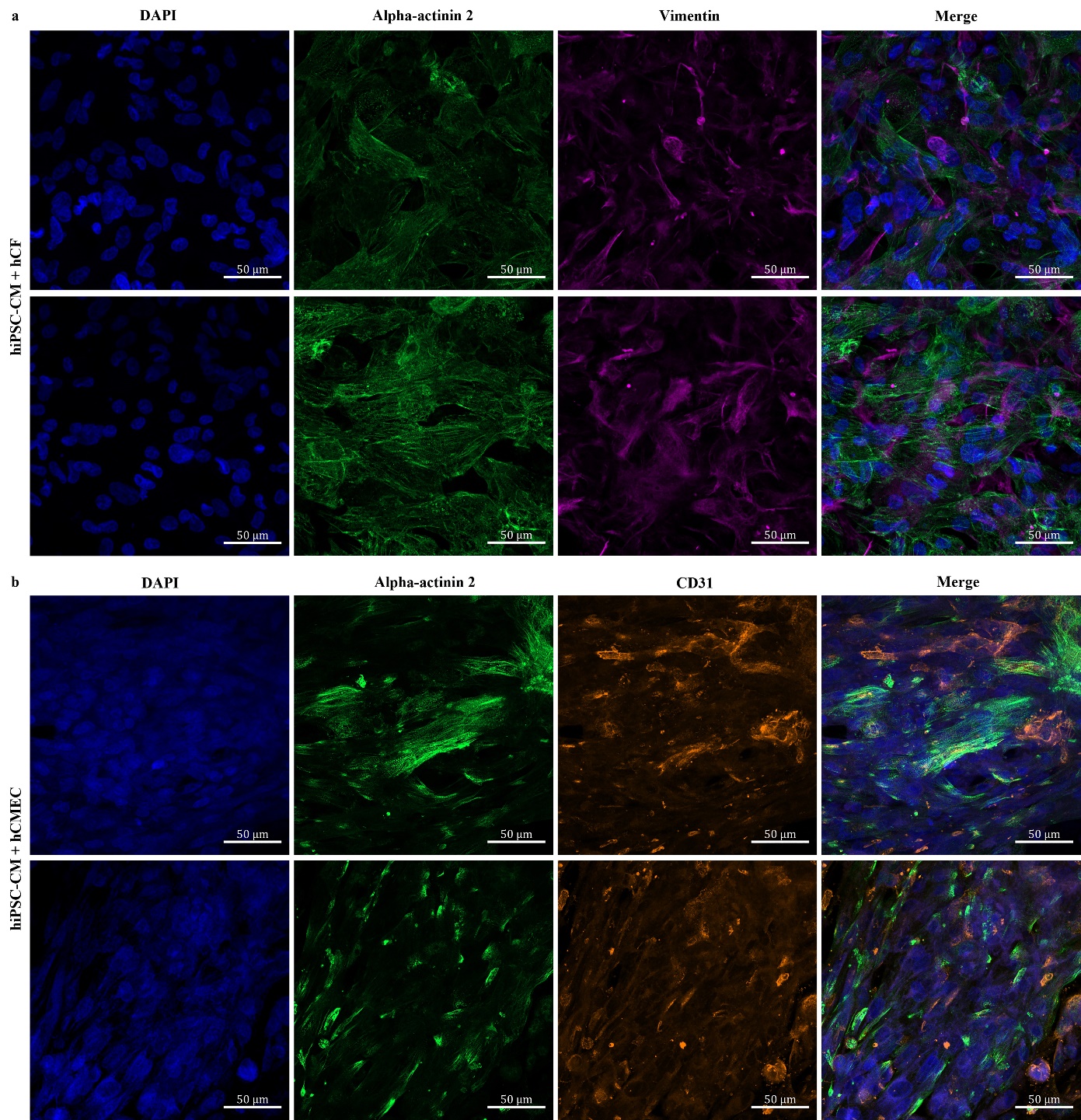


**Supplementary Figure 6.** Additional fluorescence images of cardiomyocytes co-culture with cardiac fibroblasts a) and cardiac microvascular endothelial cells b). Scale bar = 50 µm.


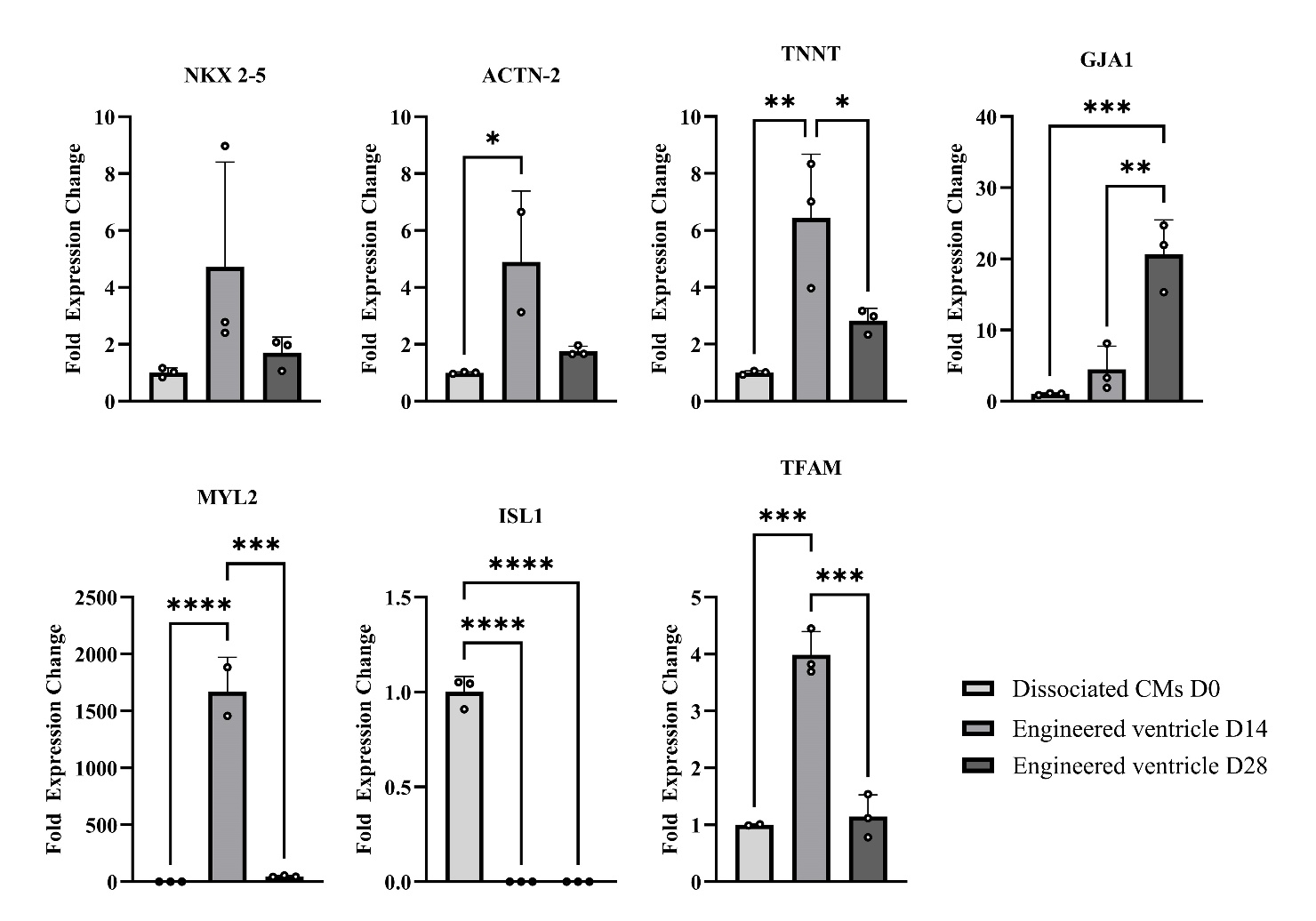


**Supplementary Figure 7:** Expression of cardiomyocyte-related genes, as well as genes associated with gap junctions and mitochondrial activity, in cardiomyocytes from engineered ventricles on day of dissociation as well as day 14 and day 28. ISL1 expression was not detected in the engineered ventricle group. Statistical analyses were performed using one-way ANOVA and n=3 technical replicates. Significance levels of p <0.05 (*), p <0.01 (**), p <0.001 (***) and p <0.0001 (****) were defined.
